# Genomics of microbial plasmids: classification and identification based on replication and transfer systems and host taxonomy

**DOI:** 10.3389/fmicb.2015.00242

**Published:** 2015-03-31

**Authors:** Masaki Shintani, Zoe K. Sanchez, Kazuhide Kimbara

**Affiliations:** ^1^Department of Applied Chemistry and Biochemical Engineering, Graduate School of Engineering, Shizuoka UniversityShizuoka, Japan; ^2^Department of Bioscience, Graduate School of Science and Technology, Shizuoka UniversityShizuoka, Japan

**Keywords:** plasmid, host, replication, conjugative transfer, Inc group

## Abstract

Plasmids are important “vehicles” for the communication of genetic information between bacteria. The exchange of plasmids transmits pathogenically and environmentally relevant traits to the host bacteria, promoting their rapid evolution and adaptation to various environments. Over the past six decades, a large number of plasmids have been identified and isolated from different microbes. With the revolution of sequencing technology, more than 4600 complete sequences of plasmids found in bacteria, archaea, and eukaryotes have been determined. The classification of a wide variety of plasmids is not only important to understand their features, host ranges, and microbial evolution but is also necessary to effectively use them as genetic tools for microbial engineering. This review summarizes the current situation of the classification of fully sequenced plasmids based on their host taxonomy and their features of replication and conjugative transfer. The majority of the fully sequenced plasmids are found in bacteria in the *Proteobacteria*, *Firmicutes*, *Spirochaetes*, *Actinobacteria*, *Cyanobacteria* and *Euryarcheota* phyla, and key features of each phylum are included. Recent advances in the identification of novel types of plasmids and plasmid transfer by culture-independent methods using samples from natural environments are also discussed.

## Introduction

Plasmids are circular or linear extrachromosomal replicons that are found in many microorganisms in the domains *Bacteria*, *Archaea*, and *Eukaryota* (Funnell and Phillips, 2004). Plasmids are transmissible by conjugation (Frost et al., 2005; Sota and Top, 2008; Frost and Koraimann, 2010). Smillie et al. (2010) reported that about 14% of the full-sequenced plasmids were predicted to be conjugative. Conjugation is one of the most effective mechanisms to spread genetic elements among bacteria (Guglielmini et al., 2011). It is therefore one of the most important “vehicles” for bacterial communication of genetic information, facilitating the rapid evolution and adaptation abilities seen in bacteria (Aminov, 2011).

**Figure 1:**
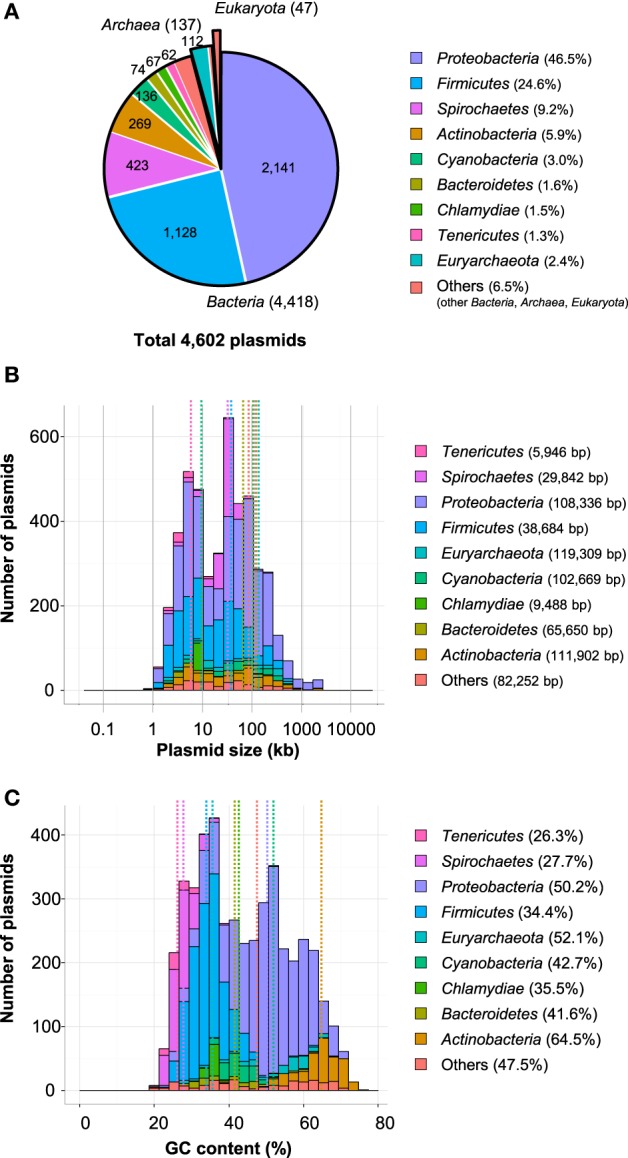
**The phylogenetic distribution of fully sequenced plasmids (A), histograms of plasmid size (B), and GC content (%) (C)**. The ratios of each phylum **(A)**, the average size of plasmids **(B)**, and the average GC contents **(C)** are shown in parentheses and as dotted lines.

Plasmids are also important genetic tools used to manipulate and analyze microorganisms through the introduction, modification or removal of target genes (Frost et al., 2005; Sota and Top, 2008). New plasmids have been reported with the recent revolution in nucleotide sequencing technology. Currently, there are 4602 complete sequences of plasmids in the NCBI Plasmid Genome database: 4418 are from bacteria, 137 are from archaea, and the remaining 47 are from eukaryota (Figure 1A; based on the NCBI database, http://ftp.ncbi.nih.gov/genomes/Plasmids, Aug. 2014).

Knowledge of the relationships between plasmid features and host taxonomy is important in order to understand how the plasmids have been spread among microbes. The classification of known plasmids is necessary to identify newly isolated plasmids in the future. Information about the host range of these plasmids is critical to effectively use them as genetic tools for microbial engineering. This information is also important to detect, isolate and identify novel types of plasmids in environmental samples.

In this review, known plasmids for which the complete sequence was available were classified by their host and their (putative) replication or transfer systems. The classification may help to predict which types of plasmids could be replicated in or be transferred to the host microbes. Several insights into the mechanisms of replication and conjugative transfer used by plasmids are summarized first, followed by a description of the features of representative plasmids in several phyla. Recent advances in the identification of novel types of plasmids and plasmid transfers by culture-independent methods using samples from natural environments are also described.

## Replication and transfer mechanisms

In plasmids, replication occurs at a specific site known as the origin of vegetative replication (*oriV*). Well-known replication systems of circular plasmids include theta-type replication, rolling-circle replication, and strand displacement-type replication (del Solar et al., 1998). Many theta-type replicating plasmids contain repeated DNA sequences, or iterons, which bind the replication initiation protein, Rep (reviewed in Espinosa et al., 2000; Krüger et al., 2004). The mechanisms of replication control have been extensively studied in two iteron-containing plasmids, pPS10 and R6K (Giraldo and Fernandez-Tresguerres, 2004; Rakowski and Filutowicz, 2013). ColE1-family plasmids are another group of theta-type replicating plasmids whose replication is strictly controlled by an antisense RNA (Polisky, 1988; Cesareni et al., 1991; Espinosa et al., 2000; Brantl, 2004). The rolling-circle replication mechanism is found in many small multi-copy plasmids (Espinosa et al., 2000; Khan, 2005). The representative plasmids using rolling-circle replication are pT181 (Inc14; see below for the discussion of Incompatibility groups), pC194 (Inc8), pMV158 (Inc11), and pUB110 (Inc13), all identified in staphylococci (Khan, 2005). Similar plasmids are also found in *Bacillus* (Guglielmetti et al., 2007) and several genera in *Actinobacteria* (Ventura et al., 2007).

Ravin recently reviewed the replication mechanisms of a prophage of *Escherichia coli*, N15, which was the first linear plasmid identified with covalently closed ends (Ravin, 2011). Most linear plasmids have conserved “telomeres” containing inverted repeat sequences (Ravin, 2011). The 5′ telomeric ends are blocked by covalently attached telomere terminal proteins (Ravin, 2011). Linear plasmids have sets of conserved telomere replication genes known as *tpg* and *tap* (Bao and Cohen, 2001, 2003), or *tpc* and *tac* (Huang et al., 2007). Many linear type plasmids have been found in *Actinobacteria*, especially in the genera *Mycobacteria*, *Rhodococcus*, and *Streptomyces* (Ventura et al., 2007).

Conjugative transfer is another important mechanism by which plasmids spread DNA among different bacteria. Self-transmissible plasmids in Gram-negative bacteria generally carry complete sets of genes required for transfer, the origin of transfer (*oriT*), the relaxase protein, the type IV coupling protein (T4CP), and the type IV secretion system (T4SS). Garcillán-Barcia et al. (2009, 2011) and Smillie et al. (2010) classified the conjugative, or mobilizable, plasmids in the GenBank database into six mobility (MOB) types (MOB_C_, MOB_F_, MOB_H_, MOB_P_, MOB_Q_, and MOB_V_) according to the amino acid sequences of their relaxase proteins. An additional classification was performed based on the plasmids' T4SS involved in mating pair formation (MPF) during conjugation. Smillie et al. proposed four classes of MPF (MPF_F_, MPF_G_, MPF_I_, and MPF_T_) according to the T4SS amino acid sequences (Smillie et al., 2010). They also investigated the presence of two key elements of plasmid mobility, type IV pili coupling protein (T4CP) and the ATPase VirB4 (Smillie et al., 2010). During conjugation, double-stranded plasmid DNA is cleaved at the *oriT* site by a relaxase protein, which then covalently binds to the *oriT* DNA. The resultant DNA-protein complex is transported to the recipient cell by T4SS. This single-stranded DNA is transferred into the recipient cell by the T4CP. The mobilizable plasmids only have *oriT*, relaxase, and sometimes T4CP (Garcillán-Barcia et al., 2009, 2011).

Gram-positive bacteria transfer plasmids by two methods, although the detailed mechanisms are not well understood. First, a single strand of plasmid DNA is transported via a T4SS, which seems to be widely used as a means for transferring plasmids in Gram positive bacteria (Goessweiner-Mohr et al., 2013). Several plasmids of the order *Actinomycetales* have conjugative systems that function in a manner similar to the segregation of chromosomal DNA during bacterial cell division and sporulation. The translocation of double-stranded DNA to the recipient cell is mediated by an FtsK-homologous protein (Goessweiner-Mohr et al., 2013). As for archaeal plasmids, only the plasmids in *Sulfolobales* are known to be transferred (Greve et al., 2004); however, the mechanisms are still not well understood.

## Distribution and classification of plasmids

Wide variations in both size and GC contents were observed among the 4602 plasmids found in the GenBank database. The average size was 80 kb (range: 744 bp-2.58 Mb), and the average GC content was 44.1% (range: 19.3–75.6%; Table S1). More than 90% of the plasmids in the database were identified in 22 phyla: *Proteobacteria* (2142 sequences, 47%), *Firmicutes* (1129 sequences, 25%), *Spirochaetes* (423 sequences, 9.2%), *Actinobacteria* (269 sequences, 5.8%), *Cyanobacteria* (136 sequences, 3.0%) *Bacteroidetes* (74 sequences, 1.6%), *Chlamydiae* (67 sequences, 1.5%), and *Tenericutes* (62 sequences, 1.3%; Figure 1A). The relationships between the phyla of plasmid hosts and plasmid size or GC content are shown in Figures 1B,C. The bimodal distribution of plasmid sizes was plotted as previously shown (Smillie et al., 2010; Garcillán-Barcia et al., 2011) with peaks at approximately 4–8 kb and 32 kb, whereas the number of sequenced plasmids increased from 1730 plasmids to 4602 plasmids. This fact indicates that the distribution of plasmid sizes is conserved if the number of sequenced plasmids increases. The average size of the 4602 plasmids (79.8 kb) was larger than those of 1730 plasmids (63.5 kb). The difference was due to the number of plasmids more than 1 Mb in 4602 plasmids (45 plasmids) was much larger than those in 1730 plasmids (9 plasmids). The average sizes of plasmids in the phyla *Actinobacteria*, *Bacteroidetes*, *Cyanobacteria*, *Proteobacteria*, and *Euryarcheota* were similarly distributed (650–1200 kb), while those in *Chlamydiae*, *Firmicutes, Spirocaetes*, and *Tenericutes* were rather small (6–40 kb, Figure 1B). However, the average GC content of plasmids was different in each host phylum (26.3–64.5%, Figure 1C). Nishida reported that the GC contents of the majority of plasmids were lower than those of their host chromosomes, although the difference was less than 10% (Nishida, 2012). Thus, it was reasonable that plasmids found in *Actinobacteria* had the highest GC content (64.5%; Figure 1C), because their host genomes ranged from 51% to greater than 70% GC (Ventura et al., 2007). It was suggested that the lower GC contents of plasmids than those of host chromosome might be due to the higher energy cost to maintain G and C than A and T (Rocha and Danchin, 2002). On the other hand, the replication of plasmids depending on the host cell's respiration machinery might be expected to have the same GC content as the host (Rocha and Danchin, 2002). Therefore, the GC content of the plasmid is likely to be important for determining its host range. It was suggested that the nucleotide composition of plasmid might be progressively altered toward the average nucleotide composition of the host genome (Lawrence and Ochman, 1998; Rocha and Danchin, 2002). Based on the hypothesis, the relationships of GC contents between the plasmid and the host genome might be also important for predicting when the plasmid was introduced into the host species. Indeed, even in the identical host species, the ranges of GC contents of plasmids were wider than those of the host genome sequence. For example, the GC contents of the whole nucleotide sequences of 10 plasmids in *Pseudomonas aeruginosa* (Table S1) ranged from 45.8 to 63.8% (the average GC contents were 58.7%). According to the *Pseudomonas* genome database (http://www.pseudomonas.com/), the GC contents of *Pseudomonas aeruginosa* ranged from 66.1 to 66.6% (average GC contents = 66.4%). The plasmid with 63.8% GC content might be introduced into strains *P. aeruginosa* earlier than the one with 45.8%.

Plasmids have been classified based on incompatibility since the 1970s. Incompatibility (Inc) is defined as the inability of plasmids sharing similar replication and partition systems to be propagated stably in the same host cell line. Inc groups have been independently classified in three different genera; there are 27 Inc groups in *Enterobacteriaceae*, 14 Inc groups of *Pseudomonas*, and approximately 18 Inc groups in *Staphylococcus* (Udo and Grubb, 1991; Lawley et al., 2004; Taylor et al., 2004; Thomas and Haines, 2004; Sota and Top, 2008; Carattoli, 2009). Several Inc groups of *Pseudomonas* are identical to those in enterobacteria, such as IncP-1 (equivalent to IncP), IncP-3 (equivalent to IncA/C), IncP-4 (equivalent to IncQ), and IncP-6(equivalent to IncG/U). Classification into an Inc group is always based on the amino acid sequence of the replication initiation (Rep) protein (replicon typing), and it is not necessarily confirmed by conventional methods whether the plasmid shows incompatibility with the same Inc group plasmid in the same host cell line. The classification based on the replicon typing is also useful for grouping the plasmids in unidentified Inc groups.

As described above, classification of plasmids based on their MOB types and MPF classes has previously been reported (Garcillán-Barcia et al., 2009, 2011; Smillie et al., 2010). Plasmid classification by replicon typing is based on the molecular characteristics of the replicons and has been quite successful (Carattoli et al., 2005). There are difficulties with this method: (i) plasmids frequently carry multiple replicons, and it is therefore difficult to classify the plasmid into single replicon group. (ii) Detailed information about Inc groups or Rep types is limited among several microbial taxonomies especially enterobacteria, and it is difficult to identify replication regions for the other types of plasmids. The classification of plasmids based on their mobility (Garcillán-Barcia et al., 2009; Smillie et al., 2010; Garcillán-Barcia et al., 2011) can overcome these problems because (i) classification by MOB types can cover the whole microbial plasmids, and (ii) plasmids rarely carry more than one relaxase gene (Garcillán-Barcia et al., 2009). However, this classification is not appropriate for non-transmissible plasmids.

**Figure 2:**
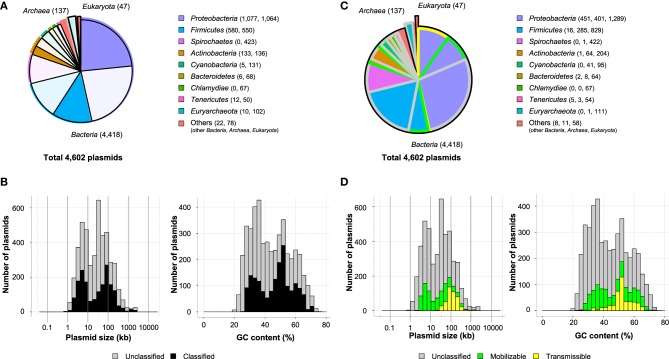
**Ratios of classified plasmids in each phylum (A) and histograms of their size (B, left) and GC content (B, right) are shown**. The unclassified plasmids are shown as shaded, and the numbers of classified plasmids and unclassified plasmids are shown in parentheses **(A)**. Ratios of putative transmissible plasmids (yellow), putative mobilizable plasmids (green), and others (gray) in each phylum **(C)** are shown. Histograms of their size (**D**, left) and GC content (**D**, right) are shown.

In this review, 4602 plasmids with complete sequences listed in the GenBank database were classified based on the genes encoding (putative) Rep proteins, MOB classes, and MPF types. Amino acid sequences of previously identified Rep proteins were used; their accession numbers in the DDBJ/EMBL/GenBank database are listed in Table S2-1. The local TBLASTN program was used for the classification of Rep proteins with the following parameters: *e*-value <10^−5^, >50% identity, and >0.5 query coverage. The parameters for identity and coverage were chosen based on variations in the amino acid sequences of the replication initiation protein TrfA from the IncP-1 plasmid (data not shown). The IncP-1 plasmid is one of the best-studied plasmids distributed across many bacterial classes (Adamczyk and Jagura-Burdzy, 2003), and thus, its TrfA protein sequence was used to set the criteria for analysis. For the ColE1 family plasmids, nucleotide sequences of RNA II were used as queries for the local BLASTN program using an *e*-value <10^−5^. Notably, the Rep proteins of the IncB, IncFII (RepA1), IncI, and IncK plasmids showed greater than 87% identity with one another. Similarly, the Rep proteins of Inc4, Inc9, Inc10, and Inc14 plasmids have 311–314 amino acid sequences and share a high degree of identity (>75%) with each other. These findings suggest that plasmids from these Inc groups are closely related to one another. In the case that one open reading frame simultaneously showed identity with the Rep proteins of these Inc groups, the one with the smaller *e*-value was used for the classification. Among the 4602 plasmids analyzed, 1845 plasmids (40.0%) in *Proteobacteria*, *Firmicutes*, *Actinobacteria*, *Cyanobacteria*, *Bacteroidetes*, *Tenericutes*, *Euryarchaeota* and other phyla were classified into previously known Inc groups or unidentified Inc groups with other known Rep types (Figures 2A,B), although several plasmids were classified into multiple Inc groups (Table S1).

The classification of plasmids into MOB classes and MPF types using T4CPs and VirB4 homologs was performed as proposed by de la Cruz's group (Garcillán-Barcia et al., 2009; Smillie et al., 2010). Instead of using the local PSI-BLAST program (ver. 2.2.24, http://ftp.ncbi.nlm.nih.gov/blast/executables/blast+/LATEST/) as described previously (Garcillán-Barcia et al., 2009), we used the local TBLASTN program with >50% identity and >0.7 query coverage for the alignment of MOB and MPF gene sequences (lists of queries are shown in Table S2-2). The MPF type of a plasmid was determined by the presence of more than two MPF genes on the plasmid. There are five MPF types of plasmids, as defined by de la Cruz's group: (i) non-transmissible plasmids that do not code for a relaxase (“non-mob”); (ii) plasmids that do not contain a relaxase but contain T4CP, VirB4, and MPF, or any two of these three elements (“non-mob, protein export”); (iii) mobilizable plasmids that contain a relaxase gene but lack VirB4 and MPF (“mob”); (iv) conjugative plasmids that contain a known type of T4SS (MPF_F_, MPF_T_, MPF_I_ or MPF_G_), plus relaxase and T4CP (“determined conjugative”); and (v) conjugative plasmids that contain genes for relaxase, T4CP, and VirB4, but whose T4SS does not belong to a specific MPF (“undetermined conjugative”). Because the relationship between the plasmid and each MOB class or MPF type was previously reviewed in detail (Garcillán-Barcia et al., 2009, 2011; Smillie et al., 2010), the distribution of “mob” or putative mobilizable, “determined conjugative” or putative transmissible, and the other plasmids were discussed in this review. As shown in Figure 2C, putative transmissible plasmids were primarily found in the phylum *Proteobacteria*, with smaller numbers found in *Firmicutes*, *Bacteroidetes*, *Tenericutes*, and others. Many putative mobilizable plasmids were found in the phyla *Proteobacteria* (401/2141 plasmids, 18.7%), *Firmicutes* (285/1130 plasmids, 25.2%), *Actinobacteria* (64/269 plasmids, 23.8%), and *Cyanobacteria* (41/136 plasmids, 30.1%) (Figure 2C). The mobilizable plasmids had a mean peak at a smaller size (around 5 kb) than that of the transmissible plasmids (around 100 kb; Figure 2D, left). The two major peaks of mobilizable and transmissible plasmids were similarly found as those in previous report (Smillie et al., 2010). This fact indicates that the size distribution of mobilizable and transmissible plasmids is conserved if the number of full-sequenced plasmids increases. As for the distribution of the GC content of the plasmids, both types showed a wide range of values (23–74%; Figure 2D, right), probably because various hosts harbor both mobilizable and conjugative plasmids.

## Plasmids in *proteobacteria*

**Figure 3:**
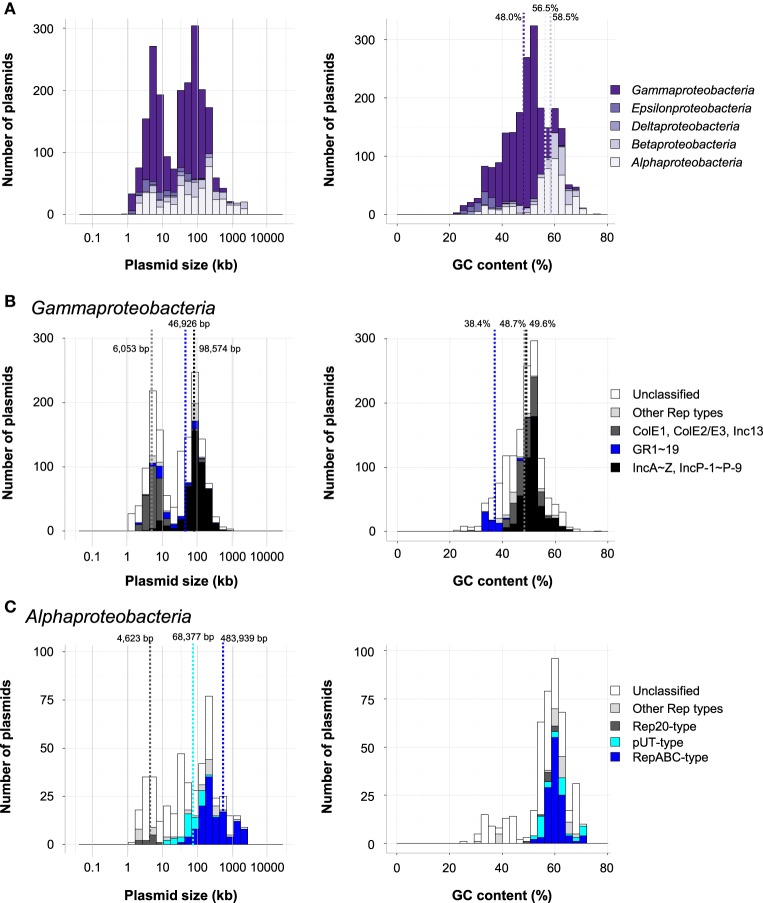
**(A)** Histograms of plasmid size (left) and GC content (right) of *Proteobacteria* in each class. **(B)** Histograms of plasmid size (left) and GC content (right) in *Gammaproteobacteria*. The distribution of classified plasmids are shown in black (IncA-Z and IncP-1 to IncP-9), blue (GR1 to GR19), dark gray (ColE1, ColE2/E3, and Inc13), light gray (other Rep types), and white (unclassified). **(C)** Histograms of plasmid size (left) and GC content (right) in *Alphaproteobacteria*. The distribution of classified plasmids are shown in blue (RepABC-type), light blue (pUT-type), dark gray (Rep20-type), light gray (other Rep types), and white (unclassified). The average sizes of Rep20-type, pUT-type, and RepABC-type plasmids are represented by dotted lines.

Plasmids within *Proteobacteria* are one of the most extensively studied groups because they include those harboring the pathogenic genes of bacteria known to infect both animals and plants. Indeed, about half of the plasmids in the database were found in *Proteobacteria* (Figure 1A). Although many catabolic plasmids have also been identified in the phylum *Proteobacteria*, they are not described in detail here, as they have been reviewed elsewhere (Shintani et al., 2010; Shintani and Nojiri, 2013). The majority of the plasmids within this phylum were found in the classes *Gammaproteobacteria* (1389; 63%), *Alphaproteobacteria* (451; 22%), and *Betaproteobacteria* (187; 8.7%). The size distribution was bimodal, with one group at around 5 kb and the other at 65–131 kb (Figure 3A, left), although the average size of plasmids in each major class was different: 58.7 kb in *Gammaproteobacteria*, 218 kb in *Alphaproteobacteria*, and 259 kb in *Betaproteobacteria*. The average GC contents of plasmids in these groups were 48.0% in *Gammaproteobacteria*, 56.5% in *Alphaproteobacteria*, and 58.5% in *Betaproteobacteria* (Figure 3A, right). Within this phylum, 1077 plasmids were classified using previously known Rep proteins.

## Plasmids in *gammaproteobacteria*

Among the 1396 plasmids in *Gammaproteobacteria*, 830 were classified into Inc groups (IncA to IncZ, IncP-1 to IncP-9, and Inc13; 485 plasmids), the ColE1-type plasmid group (210 plasmids), the ColE2/E3-type plasmid group (38 plasmids), and other groups with known Rep (119 plasmids), while several plasmids were classified into multiple Inc or Rep type groups (Table S1). The average sizes of plasmids classified into Inc13, ColE1-type, and ColE2/E3-type plasmids (6053 bp) were smaller than those of other classified plasmids (98,574 bp) (Figure 3B, left), although the average GC contents were similar between the smaller and larger groups (48.7% and 49.6%, respectively; Figure 3B, right). This was probably because the sizes of the plasmids are closely related to their replication systems.

The majority of plasmids in *Gammaproteobacteria* were from *Enterobacteriales* (927) and *Pseudomonadales* (183). The plasmids from *Enterobacteriales* contain antibiotic resistance plasmids, which cause the rapid development of resistance to antibiotics in bacterial populations. Thus, more than 75% of the plasmids in *Enterobacteriales* (697/927) were classified into Inc groups or Rep types, including IncF, IncA/C (equivalent to IncP-3), IncL/M, IncI, IncHI2/S, and IncN, containing plasmid families already known to carry resistance genes (Carattoli, 2009; Wang et al., 2013). Carattoli and Partridge have separately reviewed the emergence and spread of antibiotic resistance via plasmids among *Enterobacteriaceae* (Carattoli, 2009; Partridge, 2011). The spread of plasmids carrying genes for extended-spectrum β-lactamases (ESBLs) has been a particular problem in the treatment of *Enterobacteriaceae* infections (Paterson, 2006). Plasmid groups found in *Enterobacteriales*, such as *Enterobacter*, *Escherichia*, *Salmonella*, *Shigella*, and *Yersinia*, include a large number of virulence plasmids. It should be noted that virulence-associated traits of *E. coli* are almost exclusively found on IncF-family plasmids (Johnson and Nolan, 2009). In virulent *Yersinia* species, extensive research has been carried out on “low calcium ion (Ca^2+^) response plasmids,” which regulate the growth and expression of several virulence-associated properties by Ca^2+^ and temperature. This group includes the pCD1, pYVe8081, and pYVe227 series, which encode a set of secreted anti-host proteins and a type III secretion system (Straley et al., 1993). Notably, proteins responsible for replication initiation encoded on pCD-type plasmids showed high similarity with those encoded on IncZ plasmids, and 186 of the 976 plasmids of *Enterobacteriales* carried genes encoding these types of Rep proteins (Table S1).

Although the IncP-1 to IncP-14 groups have been found in *Pseudomonas*, only 21 plasmids in the order *Pseudomonadales* were classified into these groups; the other 93 plasmids classified into these Inc groups were in other orders (including *Enterobacteriales* and *Thiotrichales*), other classes (including *Alphaproteobacteria* and *Betaproteobacteria*), or even other phyla (including *Actinobacteria* and *Thermotogae*; Table S1). Recently, Xiong et al. determined the complete sequence of pOZ176, an IncP-2 plasmid which is resistant to heavy metals (Xiong et al., 2013). Ten additional plasmids have been identified which carry a gene encoding a putative Rep protein with >50% identity and >0.5 query coverage, suggesting that these plasmids may have IncP-2-like replication systems (Table S1). The IncP-2 group contains degradative plasmids whose sizes are typically around 500 kb (Shintani et al., 2010; Shintani and Nojiri, 2013); the average size of the ten plasmids was 425 kb (Table S1). Two plasmids were found to share >83% identity with the sequence of the Rep protein from pOZ176: pPNAP01, a plasmid of *Polaromonas naphthalenivorans* CJ2, encodes proteins involved in the degradation of naphthalene (Jeon et al., 2003, 2006; Yagi et al., 2009), and pBB1 (GenBank/EMBL/DDBJ accession no. CP002879), a plasmid of *Cupriavidus necator* N-1, encodes proteins putatively involved in the degradation of catechols (Poehlein et al., 2011). Taken together, these findings suggest that the nucleotide sequences of the replication systems used by previously-known Inc group plasmids could be important tools for classifying other plasmids. It will be therefore helpful for the classification to determine the nucleotide sequences of the other unsequenced Inc group plasmids including IncP-5, IncP-8, IncP-10, IncP-11, IncP-12, IncP-13, and IncP-14 plasmids (Table S2-1).

*Acinetobacter baumannii* is known to be an important pathogen, and the plasmids in the genus *Acinetobacter* are considered to be key genetic factors in the spread of multi-drug resistance (Dijkshoorn et al., 2007; Evans and Amyes, 2014). Bertini et al. proposed PCR-based replicon typing of 19 groups (GRs) of resistance plasmids in *A. baumannii* on the basis of nucleotide sequence similarities between their replication initiation proteins (Bertini et al., 2010). Using the amino acid sequences of Rep from plasmids in the GR1 to GR19 groups, we were able to classify 78 of the 4602 plasmids found in the database, including 60 of the 90 plasmids in genus *Acinetobacter* (Table S1). The remaining 18 plasmids were also found in *Gammaproteobacteria*, and, notably, these plasmids were all classified into GR3 (Table S1). The average GC contents of plasmids classified into GR1-19 (38.4%) were lower than those of other plasmids in *Gammaproteobacteria* (Figure 3B). These findings indicate that the plasmids with GR1- to GR19-type Rep proteins were primarily distributed in *Acinetobacter* and that GR3 group plasmids may have a broader host range than plasmids in other GR groups.

## Plasmids in *alphaproteobacteria*

The majority of the 451 plasmids in *Alphaproteobacteria* were found in *Rhizobiales* (143 plasmids), *Rhodospirillales* (122 plasmids), *Rhodobacteriales* (94 plasmids), and *Sphingomonadales* (65 plasmids). Many plasmids in *Alphaproteobacteria* carry genes encoding RepABC proteins (Cevallos et al., 2008). The RepAB proteins are involved in the partitioning of the plasmid, and RepC is associated with replication initiation (Cevallos et al., 2008; Pinto et al., 2012). Indeed, 123 plasmids in *Alphaproteobacteria* had RepABC type genes (Table S1). Although plasmids containing these types of Rep genes have an average size of 484 kb, their sizes vary widely from 30 to 2430 kb (Figure 3C).

Hosts in the *Rhizobiales* are known to be symbiotic bacteria, and they can usually fix nitrogen only upon establishing a mutualistic interaction with plants, particularly those of the *Leguminosae* family (Cevallos et al., 2008; Pinto et al., 2012). Their 143 plasmids are characterized by a highly variable plasmid number (from 0 to 11) and size (from 150 to 1683 kb). Of these, 101 plasmids were classified into the RepABC type group (Table S1). Notably, most of the genes for nodulation and nitrogen fixation are located on a single plasmid, called the symbiotic plasmid (pSym). pSym contains the structural genes for nitrogenase (*nifHDK*) and/or genes essential to the production of the nodulation factor (*notABC*). Introduction of pSym into a plasmid-free *A. tumefaciens* strain leads to nodulation of a specific host and, sometimes, to nitrogen fixation, albeit at modest levels. Conversely, elimination of pSym impairs both the nodulation and nitrogen fixation capacities of the original bacterial strain (Torres et al., 2011). The presence of pSym is common to members of the fast-growing *Rhizobium* and *Sinorhizobium* species, including symbionts of many plants of agronomic interest. RepABC plasmids were also found in classes *Rhodospirillales* and *Rhodobacteriales* (Table S1) as previously reported (Petersen et al., 2011).

Approximately half of the plasmids in class *Rhodosprillales* were found in *Acetobacter* (60 of 122 plasmids; Table S1). The acetic acid bacteria are important for the industrial oxidation of various compounds, and thus, the construction of shuttle vector(s) to facilitate functional gene studies of these bacteria was reported 30 years ago (Fukaya et al., 1985; Okumura et al., 1985). Nevertheless, a well-defined method for the classification of *Acetobacter* plasmids has not been developed. Two groups have recently reported the characterization of Rep proteins, Rep7 and Rep20, from small plasmids pGR7 (Grones and Grones, 2012) and pAG20 (Babic et al., 2014), respectively, found in *Acetobacter* (class *Rhodospirillales*). The Rep20 gene was also found in plasmids in *Rhodospirillales* and *Rhodobacteriales* (Figure 3C). The average size of these plasmids was 4.6 kb, while that of RepABC-type plasmids was 483 kb (Figure 3C), suggesting that the newly characterized Rep proteins may be appropriate for the replication of small plasmids.

Several plasmids in *Sphingomonadales* have been found in bacteria which degrade biphenyl (pNL1 and pNL2 in *Novosphingobium aromaticivorans* DSM 12444; (Stillwell et al., 1995; Romine et al., 1999), gamma-hexachlorocyclohexane (pCHQ1, pUT1, and pUT2 in *Sphingobium japonicum* UT26S; (Nagata et al., 2006, 2010, 2011), and dibenzo-*p*-dioxin (pSWIT01 and pSWIT02 in *Sphingomonas wittichii* RW1; (Miller et al., 2010). The (putative) Rep proteins of these plasmids (pCHQ1, pUT1, pUT2, pSWIT01, pNL1, and pNL2) were found in 59 other plasmids in *Alphaproteobacteria*, and 24 plasmids were found in *Sphingomonadales* (Table S1). Notably, 42 plasmids in *Alphaproteobacteria* were classified as pUT1- or pUT2 type plasmids, and 36 of these were the pUT1 type (Figure 3C). The average size of the pUT-type plasmids was 68.4 kb, which was larger than Rep20 type plasmids and smaller than RepABC-type plasmids (Figure 3C). Most of the plasmids classified into these three Rep types were in *Alphaproteobacteria* (Table S1), whereas two pUT1-type plasmids and six RepABC-type plasmids were found in other classes or phyla (Table S1). Thus, they are important vehicles among *Alphaproteobacteria*, although the plasmids with smaller sizes and lower GC contents were still unable to be classified (Figure 3C).

## Plasmids in *firmicutes*

**Figure 4:**
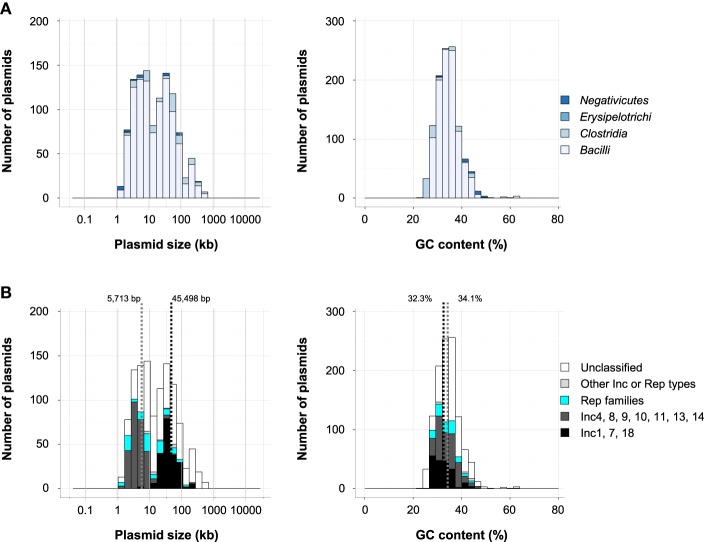
**(A)** Histograms of plasmid size (left) and GC content (right) of *Firmicutes* in each class. **(B)** Histograms of plasmid size (left) and GC content (right) in *Bacilli*. The distribution of classified plasmids is shown in black (Inc1, Inc7, and Inc18), dark gray (Inc4, Inc8, Inc9, Inc10, Inc11, Inc13, and Inc14), light blue (Rep families number 1–24 containing 7b, 10b), light gray (other Inc or Rep types) and white (unclassified). The average sizes and GC contents of groups Inc4, 8, 9, 10, 11, 13, 14 and those of Inc1, 7, 18 are represented by dotted lines.

Plasmids found in *Firmicutes* were smaller (1.3–627 kb, 39 kb average) and had lower GC contents (23–63%, 34.4% average) than those in *Proteobacteria* (0.74–2580 kb, 103 kb average; 22–76%, 50.2% average; Figures 1B,C). Among the 1129 plasmids in *Firmicutes*, 1021 plasmids, or 90%, were found in the class *Bacilli* (Figure 4A). Based on comparisons with the amino acids sequences of (putative) Rep genes of the Inc groups of staphylococci, 475 plasmids in *Firmicutes* were shown to have putative Rep genes of Inc1 to Inc18 plasmids (Figure 4B), and 311 plasmids were from *Bacilliales*, including *Bacillus* (57 plasmids) and *Staphylococcus* (220 plasmids; Table S1). A subset of 158 plasmids classified into Inc1 to Inc18 groups was found in *Lactobacillales*, including *Enterococcus* (36 plasmids), *Lactobacilllus* (56 plasmids), *Lactococcus* (15 plasmids), and *Streptococcus* (34 plasmids; Table S1). The Rep genes of the Inc4, Inc8, Inc9, Inc10, Inc11, Inc13 and Inc14 group plasmids are known as rolling-circle replication plasmids (Smith and Thomas, 2004; Khan, 2005; Guglielmetti et al., 2007), while those of Inc1, Inc7, and Inc18 are theta-type replication plasmids (Bruand et al., 1991; Jensen et al., 2010b; Liu et al., 2013). Another classification system based on the nucleotide sequences of plasmids was proposed for enterococci and staphylococci, which included 26 Rep families and 10 unique families (Jensen et al., 2010a; Lozano et al., 2012). Of the 4602 plasmids included in our analysis, 388 plasmids could be classified using the 26 Rep families, and 383 of them were found in *Firmicutes* (Table S1). Several plasmids were classified both a Rep family and an Inc group, but 91 plasmids were only classified into the Rep family (Figure 4B). The distribution of plasmid size was bimodal, and the 269 putative rolling-circle replication plasmids had a smaller average size (5713 bp), while the average size of the 200 theta-type replication plasmids was larger (45,498 bp; Figure 4B, left). The GC contents of both groups were similar, 32.3% and 34.1%, respectively (Figure 4B, right).

Plasmids conferring resistance and virulence to *Bacilli*, including *Enterococcus* (*Bacilliales*) and *Staphylococcus* (*Lactobacilliales*), are causative agents of hospital infection outbreaks. The transmission of antibiotic resistance to *Enterococcus* is mediated by the pheromone-responsiveness and broad host range of Inc18 group plasmids (Palmer et al., 2010). The pheromone-responsive plasmids have mainly been described in *Enterococcus faecalis*. The representative examples of these plasmids are pCF10 and pAD1 (Clewell, 2007; Dunny, 2007). Their transfers are induced by pheromones generated from lipoprotein signal peptides encoded on the host chromosome (Clewell, 2007; Dunny, 2007). Pheromone receptors are encoded on the plasmid, and binding of the pheromone to its receptor causes an effective pair formation between donor and recipient cells. Rosvoll *et al*. have reported an in-depth investigation of Inc18 plasmids, which are found mostly in *E. faecium* (Rosvoll et al., 2010). Of these, only pAMβ1 and pIP501 have been well-characterized and were originally identified in *E. faecalis* and *Streptococcus agalactiae*, respectively. Within *Staphylococcus*, the pSK1 family (Inc1, Rep 20 family), which includes a multidrug-resistance plasmid like pSK4 and pSK7, and the pSK41 family (Inc7, Rep 15 family), including a conjugative multidrug-resistance plasmid like pGO1, have been investigated along with the previously discussed rolling-circle replicating plasmid (Jensen et al., 2010b; Liu et al., 2013).

The majority of the lactic acid bacteria (LAB) that belong to *Bacilli*, including *Lactococcus*, *Lactobacillus*, *Leuconostoc*, *Pediococcus*, and *Streptococcus*, as well as *Bifidobacterium* (phylum *Actinobacteria*), are important in food fermentation. Recently, the plasmids from *Lactococcus lactis* were reviewed in detail regarding plasmid-encoded traits of biotechnological significance (Ainsworth et al., 2014). Genetic engineering technology, including the development of cloning vectors for the LAB, is critical for the effective application of these bacteria in the food industry (Shareck et al., 2004). Versatile vectors have been developed for high-level, inducible gene expression in *Lactobacillus sakei* and *Lactobacillus plantarum* (Sorvig et al., 2005). In the GenBank database, 166 plasmids were found in *Lactobacillus*, and notably, multiple (five to ten) plasmids were found in individual strains, such as *Lactobacillus fermentum* MTCC 8711 (Jayashree et al., 2013), *Lactobacillus plantarum* 16 (Crowley et al., 2013), and *Lactobacillus reuteri* I5007 (Hou et al., 2014). Fukao et al. reported that one of the LAB, *L. brevis* KB290, has nine plasmids with RepABC systems (Fukao et al., 2013). Understanding how these plasmids could be maintained simultaneously in a single host cell will be important to the development of genetic tools for LAB.

## Plasmids in *spirochaetes*

Although 423 plasmids were found in *Spirochaetes* (Figure 1A), the majority of them were identified in genus *Borrelia* (416 out of 423), which is known to be an important pathogenic bacterium. The average GC contents of these plasmids were comparatively low (27.7%; Figure 1C). A unique feature of *Borrelia* is its segmented genome consisting of multiple circular and linear plasmids in addition to its linear chromosome within a single cell (Casjens et al., 2000). The features of *Borrelia* have been studied in detail (Chaconas and Kobryn, 2010; Chaconas and Norris, 2013). *B. burgdorferi* type strain B31, the causative agent of Lyme disease, possesses twelve linear plasmids and nine circular plasmids (Fraser et al., 1997; Casjens et al., 2000). Tilly et al. recently characterized the indispensable elements involved in the maintenance of cp26, a 26-kb circular plasmid found in *B. burgdorferi* (Tilly et al., 2012). Most plasmids can be lost without affecting the host growth; however, cp26 remains in all isolates of *B. burgdorferi* (Tilly et al., 2012). This plasmid carries genes required for survival of its host, including the *resT* gene, which encodes a telomere resolvase involved in the resolution of the replicated telomeres of the linear chromosome and plasmids in *B. burgdorferi* (Tilly et al., 2012).

Notably, several circular plasmids in B31 are homologous throughout almost their entire lengths (cp32s) and carry many genes encoding lipoproteins located on cell surfaces, although very few of these are metabolic or housekeeping genes (Casjens et al., 2000). Bunikis et al. proposed an efficient method to identify each plasmid based on multiplex PCR (Bunikis et al., 2011). The mechanisms used by the host strain to replicate and maintain similar plasmids have been studied in one of the cp32 plasmids (Eggers et al., 2002). There are still many interesting characteristics that require further investigation of plasmids in *Spirochetes*, and comparative genomic analyses of *Borrelia* in particular will reveal important information. Casjens et al. compared the genomic structures of plasmids identified in four different *Borrelia* strains (Casjens et al., 2012). They suggested that there are at least 28 plasmid compatibility types among the four strains and that several inter-plasmid genomic rearrangements may have occurred. More genomic information will be necessary for understanding the genetic evolution of these plasmids and their host chromosome.

## Plasmids in *actinobacteria*

**Figure 5:**
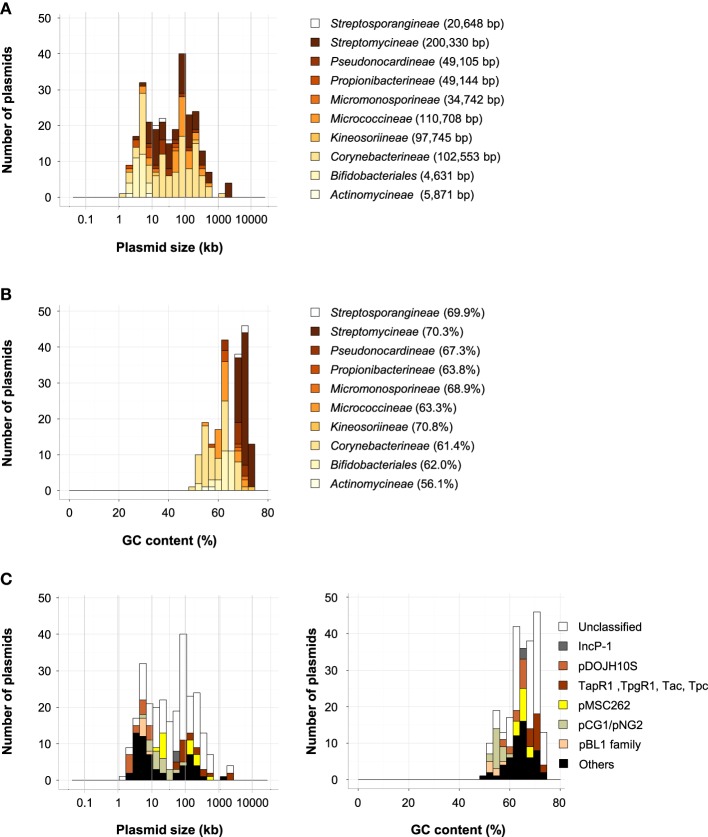
**Histograms of plasmid size (A) and GC content (B) of *Actinobacteria* in each suborder and order (*Bifidobacteriales*)**. The average size of plasmids **(A)** and the average GC content **(B)** are shown in parentheses. **(C)** Histograms of plasmid size (left) and GC content (right) in phylum *Actinobacteria*. The distribution of classified plasmids is shown in beige (pBL1 family), spring green (pCG1/pNG2 family), yellow (pMSC262 family), brown (plasmids with TapR1, TpgR1 Tac, and/or Tpc), light brown (pDOJH10S-type), and gray (IncP-1 plasmids), black (other Rep types), and white (unclassified plasmids).

All of the plasmids in the phylum *Actinobacteria* were found in class *Actinobacteria*, and, as shown in Figures 5A,B, they were found in nine suborders and one order (*Bifidobacteriales*). The plasmids in *Actinobacteria* were large and had high GC contents (Figures 1B,C). The phylum *Actinobacteria* includes a wide variety of bacteria with different morphologies, physiologies, and metabolic properties, and they have various kinds of plasmids (Ventura et al., 2007). In a review of the genomics of *Actinobacteria*, Ventura et al. showed the phylogenetic relationships between Rep proteins from actinobacterial plasmids (Ventura et al., 2007). In this review, sequences from 42 different types of Rep proteins from actinobacterial plasmids were used as queries, and of these proteins, 27 were found in more than two plasmids (Table S2-1). Of the 269 plasmids in *Actinobacteria*, 110 were found in *Corynebacterineae*, 69 in *Streptomycineae*, 32 in *Micrococcineae*, and 29 in *Bifidobacteriales*. The average sizes and GC contents of these plasmids varied widely (4631–200,330 bp; 56.1–70.3%; Figures 5A,B).

The majority of the plasmids from *Corynebacterineae* were found in *Corynebacterium* (38 plasmids), *Mycobacterium* (29 plasmids), and *Rhodococcus* (31 plasmids). Because *C. glutamicum* is widely used as an industrial producer of amino acids, plasmids of the amino acid-producing isolates were classified to develop efficient recombinant DNA techniques (Tauch et al., 2003a). Several rolling-circle replication plasmids were found in *C. glutamicum*, and the pBL1 (including pAG3 and pCG2) and pCG1 (including pCG4 and pGA2) families were proposed (Tauch et al., 2003a). A plasmid found in the human pathogen *C. diphtheria* S601, pNG2, has a Rep protein similar to those of the pCG1 family (Tauch et al., 2003a,b).

There were six pBL1-family plasmids and 22 pCG1/pNG2 plasmids in the GenBank database (Table S1). While plasmids in the pBL1 family had small sizes (4.4–6.8 kb), those in the pCG1/pNG2 family had a wide range of sizes (4.1–85 kb, Figure 5C). Tauch et al. also reported a theta-type replication plasmid family in *Corynebacterium*, the pCRY4 family, but among the 4602 plasmids analyzed, only one plasmid was classified into this family (Table S1). Of the plasmids found in *Mycobacterium*, only the low copy number plasmid pAL5000 has been widely used (Labidi et al., 1985). Plasmids in the pMSC262 family are compatible with pAL5000, including pVT2, pMUM001, and linear plasmid pCLP (Le Dantec et al., 2001), which had a wide range of sizes (24–615 kb, Figure 5C). Among the 29 plasmids of *Mycobacterium*, 16 plasmids were classified into the pMSC262 family (Table S1). Plasmids in *Rhodococcus* were found in the biphenyl degrader, *R. jostii* RHA1 (Seto et al., 1995). This strain has three plasmids, pRHL1, pRHL2, and pRHL3 (Masai et al., 1997; Shimizu et al., 2001; McLeod et al., 2006). The *R. erythropolis* PR4 strain is known to utilize n-alkanes and alkylbenzene, and it has three plasmids, pREL1, pREC1, and pREC2 (Sekine et al., 2006). Only a small number of plasmids in *Rhodococcus* were predicted to have Rep proteins similar to those of pRHL1, pRHL3, pREC1, or pREC2 (Table S1).

Plasmids in *Streptomycieae* are found in *Streptomyces* species, and several of them are large linear plasmids. These linear plasmids have conserved “telomeres” containing inverted repeat sequences (Chen et al., 1993; Pandza et al., 1998). The 5′ telomeric ends are blocked by covalently attached telomere terminal proteins (Bao and Cohen, 2001). They have sets of conserved telomere replication genes known as *tpgR1* and *tapR1* or *tpc* and *tac* (Bao and Cohen, 2001, 2003). In *Streptomyces*, 13 plasmids, all linear, carry these telomere replication gene sets (Table S1). Their sizes were relatively large (54–1797 kb, Figure 5C) and their GC contents were high (68.3–71.9%, Figure 5C). A rolling circle replication plasmid, pIJ101, from *S. lividans* has been used as a cloning vector in *Streptomyces* (Kendall and Cohen, 1988; Ventura et al., 2007), but only two other plasmids have been found to carry a similar Rep gene (Table S1).

Plasmids in *Bifidobacteriales* were small, with an average size of 4.6 kb (Figure 5A). Among the 29 plasmids in *Bifidobacteriales*, 28 of them were classified into the known Rep types pKJ50 (Park et al., 1999), pNAC2, pNAC3 (Corneau et al., 2004), and pDOJH10S (Lee and O'Sullivan, 2006). Rep proteins of pKJ50, pNAC2, and pNAC3 were found only in *Bifidobacterium*; however, those of pDOJH10S were found in plasmids of other suborders of *Actinobacteria*, including *Corynebacterineae*, *Micrococcinaea*, and *Propionibacterineae* (Table S1). It is likely that plasmids with Rep types of pDOJH10S have a broad host range in the phylum *Actinobacteria*.

## Plasmids in *cyanobacteria*

*Cyanobacteria* are known for the ability to undergo oxygenic photosynthesis and are a promising platform for the production of renewable chemicals and fuels. Thus, a plasmid vector to introduce exogenous genes into cyanobacterial cells has been developed. Taton et al. have developed a vector system for plasmids with a broad host range using the previously known plasmid origin of *Cyanobacteria* (http://golden.ucsd.edu/CyanoVECTOR/) (Taton et al., 2014). The origins of plasmids were from pDU1 in *Nostoc* sp. PCC 7524 (*Anabaena* sp. PCC 7120; Walton et al., 1992), pANS (pUH24) in *Synechococus elongatus* PCC 7942 (Golden and Sherman, 1983), pDC1 in *Nostoc* sp. PCC 8009 (*Nostoc* sp. ATCC 29133; Lambert and Carr, 1983), and pFDA in *Fremyella diplosiphon* PCC 7601 (Cobley et al., 1993). Although vectors containing these origins could be a useful module to construct new vectors for *Cyanobacteria*, the homologous rep genes were not found in other plasmids in our database. This finding indicates that *Cyanobacteria* may contain a wider variety of plasmids than other phyla. Smillie et al. (2010) proposed that cyanobacteria may use an as-yet-uncharacterized system to conjugate because, within the phylum, the plasmids encoding relaxase and/or T4CP did not possess known T4SS or VirB4 (Table S1). Indeed, plasmid transfer from *Proteobacteria* to *Cyanobacteria* has been previously reported (Encinas et al., 2014). Further in-depth analyses and classification of *Cyanobacteria* plasmids are necessary to understand the spread of plasmids in this phylum.

## Plasmids in *archaea*

Among the 4602 plasmids in the database, 137 plasmids were found in *Archaea*: 112 plasmids were in *Euryarcheoata* (Figure 1A) and 23 plasmids were in *Crenarchaeota*. There were large differences in the average sizes and GC contents between the two phyla. The plasmids in *Euryarcheoata* had an average size of 119 kb and GC content of 52.1%, while those in *Crenarchaeota* were 20 kb and 39.2%, respectively. *Euryarcheoata* includes all methanogens, haloarchaea, and some hyperthermophilic genera such as *Pyrococcus* and *Thermococcus*. Most known archaeal plasmids have cryptic phenotypes. The best characterized plasmids among methanogens are pME2001, pURB500, and pC2A (Greve et al., 2004). The putative Rep genes have been reported for pME2001 (Luo et al., 2012) and pURB500 (Tumbula et al., 1997), although multiple regions (or genes) were required for replication of pURB500. The putative gene involved in plasmid replication identified in pC2A showed limited homology with Rep proteins encoded by rolling-circle plasmids (del Solar et al., 1993; Metcalf et al., 1997). In *Thermococcales*, there are two well-characterized plasmids, pGT5 from *Pyrococcus abyssi* and pTN1 from *Thermococcus nautilus*, and their respective replication initiation proteins have been identified. Both pGT5 and pTN1 have been shown to use the rolling circle replication system (Arnold et al., 1999; Soler et al., 2007). The majority of plasmids in *Crenarchaeota* were found in *Sulfolobales* plasmids (21/23 plasmids), and they were classified into pRN-type and pNOB-type groups (Greve et al., 2004). Joshua *et al*. have reported the functional characterization of pRN1 (Joshua et al., 2013). A pRN-type plasmid, pSSVx, is known to be a virus-plasmid hybrid that coexists intracellularly with the fusellovirus SSV1 and can be packaged into viral particles (Arnold et al., 1999). The pNOB8-type plasmids are larger in size (around 30 kb) than pRN-type plasmids (less than 10 kb) and are known to be conjugative (She et al., 1998). The putative Rep gene for the pNOB8-type group has not been identified (Greve et al., 2004). Based on the TBLASTN analysis, these Rep genes of pRUB500, pME2001, pC2A, pGT5, pTN1, and pRN1 were found in only 15 plasmids of *Archaea* (Tables S1 and S2), suggesting that a wider variety of plasmids may exist in this kingdom than in others.

Genetic tools used in the analysis of archaea are currently being developed. Many archaeal species are resistant to conventional antibiotics, a factor that limits the use of genetic manipulation in archaea (Atomi et al., 2012). Several shuttle vectors are available for certain genera within *Euryarcheoata*, such as halophiles (*Haloarcula*), methanogens (*Methaonococcus* and *Methonosarcina*), and *Thermococcales* (*Pyrococcus*). However, those specific to *Crenarcheota* are only useful for the genus *Sulfolobales*. Methanogens are known to generate methane and were the first microorganisms identified as *Archaea*. Halophiles are the *Archaea* living in the most saline environments on the earth, while *Thermococcales* are found in high temperature environments, mostly above 80°C (Heuer and Smalla, 2012). *Sulfolobales* are the only members of *Crenarcheota* whose genetic manipulation methods have been established thus far (Leigh et al., 2011; Atomi et al., 2012). The plasmids of *Archaea* are typically introduced by transformation; conjugative transfer has not been reported with the exception of a small number of plasmids in *Sulfolobus* (Greve et al., 2004; Smillie et al., 2010).

## Recent work to identify other environmental plasmids

A large number of plasmids have been identified by chance during the analysis of the host bacteria based on specific phenotypes. Indeed, in our recent report of the whole genome sequence of a biphenyl-degrading bacterium, *Geobacillus* sp. JF8, we showed that it carried a plasmid, pBt40, with biphenyl-degradative genes (Shintani et al., 2014b). The putative Rep gene of pBt40 was identified, and its homologs were recently found in plasmids isolated from other strains within the genus *Geobacillus*, although detailed characterizations of these plasmids have not been performed (Table S1). Considering that most environmental bacteria cannot be cultivated, culture-dependent methods to detect transconjugants of plasmids are known to be highly biased. Smalla and Sobecky (2002) proposed multiphasic approaches for the characterization of plasmids and other mobile genetic elements (Smalla and Sobecky, 2002). Culture-independent methods have recently been developed to isolate and identify novel types of plasmids and to detect plasmid transfers (Heuer and Smalla, 2012).

## Methods for plasmid isolation and identification

Notably, 28 plasmids in the database have been identified by culture-independent methods from unidentified host strains or uncultivated microbes (Table S1). PCR using plasmid-specific sequences is one of the most common methods used to detect plasmids in environmental samples. Various primers and PCR methods to identify plasmids have been designed, such as those for *Enterobacteriaceae* (Carattoli et al., 2005), enterococci (Rosvoll et al., 2010), and other Gram-positive bacteria, mostly staphylococci (Clewell, 2007; Jensen et al., 2010a; Lozano et al., 2012). Methods of direct detection by PCR using degenerate primers have been reported for IncP-1, IncP-7, and IncP-9 plasmids from environmental samples (Dealtry et al., 2014) and for plasmids of different MOB families (Alvarado et al., 2012). Many plasmids have been identified from environmental samples, including the human gut, by metagenomic analyses (Elsaied et al., 2011; Kristiansson et al., 2011; Zhang et al., 2011; Brolund et al., 2013; Song et al., 2013). Brown Kav et al. characterized the overall plasmid population in the bovine rumen (termed rumen plasmidomes) by sequencing the extracted circular plasmids (Brown Kav et al., 2012, 2013). They developed a bioinformatics pipeline that could successfully detect low abundance plasmids and remove contamination of chromosomal DNA (Brown Kav et al., 2012). Sentchilo et al. compared the plasmid metagenomes from two separate activated sludge systems and found that the plasmids from the two environments were strongly different (Sentchilo et al., 2013). Jorgensen et al. reported an *in silico* procedure for identifying small plasmids in metagenomics datasets of the rat cecum (Jorgensen et al., 2014). They successfully identified 160 circular sequences carrying a gene with a plasmid replication domain, and the majority of these sequences were novel (Jorgensen et al., 2014).

Exogenous plasmid isolation is a culture-independent method used to capture plasmids directly from microbial communities. This method is based on the ability of the plasmid mobility and replication in a recipient. Transconjugants are selected by their phenotypes, such as antibiotic resistances, conferred by plasmid carriage. This method enables researchers to search for plasmids in a wider microcosm containing the uncultivated microbial fraction (Heuer et al., 2012). Therefore, the potential to obtain novel types of plasmids is higher. Indeed, this method has been used to isolate a number of novel plasmids (Miyazaki et al., 2006; Sobecky and Hazen, 2009; Sen et al., 2011; Eikmeyer et al., 2012; Brown et al., 2013; Oliveira et al., 2013; Norberg et al., 2014). A transposon-aided capture (TRACA) method has been developed to study and isolate plasmids independent of plasmid-encoded traits in various bacterial habitats (Jones and Marchesi, 2007). This method has also facilitated the successful identification of several novel types of plasmids (Jones and Marchesi, 2007; Zhang et al., 2011; Burmolle et al., 2012).

## Methods to detect plasmid transfer

There are multiple reviews of the conventional methods to detect plasmid transfer in various environments based on culture-dependent approaches (Smalla and Sobecky, 2002; Heuer and Smalla, 2007, 2012; Shintani et al., 2010). Systems able to directly detect plasmid transfers by culture-independent methods have been developed using fluorescent protein markers, such as GFP, DsRed, mCherry, GusA,LuxAB, with antibiotic resistance genes (Amann and Fuchs, 2008). Fluorescence *in situ* hybridization (FISH) using specific probes targeting 16S rRNA sequences and *in situ* PCR in transconjugant cells are able to detect transconjugants at the single-cell level (Amann and Fuchs, 2008; Cenciarini-Borde et al., 2009; Wagner and Haider, 2012). Fluorescent cells are also detectable and separable with the use of flow cytometry or a micromanipulator at the single cell level; both methods have a strong potential to identify actual host ranges of plasmids (Musovic et al., 2010; Shintani et al., 2014a).

*In silico* analyses have been developed to identify horizontal gene transfer phenomena including plasmid transfer. Yamashita et al. carried out a plasmidome network analysis of all available complete bacterial plasmids to identify and characterize the most recent horizontal gene transfer or plasmid transfer (Yamashita et al., 2014). de Been *et al*. reported a novel approach for the reconstruction of mobile genetic elements from whole-genome sequence data, with which they discovered specific plasmid lineages shared between farm animals and humans (de Been et al., 2014).

Over the next decade, the number of fully-sequenced plasmids will greatly increase through the use of deep sequencing methods and *in silico* analyses of datasets. The classification methods using Rep genes and mobility types based on their nucleotide sequences are useful to identify newly isolated plasmids. In this review, it is discussed genes for replication and conjugative transfer, but not the other gene(s) on plasmids including genes for resistance to antibiotics, metabolism of natural and synthetic compounds, pathogenicity, and host symbiosis. The classification lists in this review will be helpful to understand how these “other” genes on plasmids could be spread among microbes. The classification will be also of help to predict host candidates of plasmids found by a metagenomics approach, rather than mostly a collection of results obtained.

Considering that less than half of the plasmids in the database were able to be classified, there may still be unknown or novel types of plasmid replication or transfer systems in microbes. Therefore, characterizations of plasmid features involved in replication, maintenance, transfer, and host range based on molecular biological and biochemical methods are still necessary. Culture-independent methods to detect plasmids and their transfers are currently available for analyzing non-culturable or uncultivated microbes. These studies will not only enable us to understand the evolution of microbes mediated by plasmid transfers but will also provide us with useful tools for genetic analyses of both culturable and non-culturable microbes.

### Conflict of interest statement

The authors declare that the research was conducted in the absence of any commercial or financial relationships that could be construed as a potential conflict of interest.

## References

[B1] Adamczyk M. Jagura-Burdzy G. (2003). Spread and survival of promiscuous IncP-1 plasmids. Acta Biochim. Pol. 50, 425–453. 12833168

[B2] Ainsworth S. Stockdale S. Bottacini F. Mahony J. van Sinderen D. (2014). The *Lactococcus lactis* plasmidome: much learnt, yet still lots to discover. FEMS Microbiol. Rev. 38, 1066–1088. 10.1111/1574-6976.12074 24861818

[B3] Alvarado A. Garcillan-Barcia M. P. de la Cruz F. (2012). A degenerate primer MOB typing (DPMT) method to classify gamma-proteobacterial plasmids in clinical and environmental settings. PLoS ONE 7:e40438. 10.1371/journal.pone.0040438 22792321 PMC3394729

[B4] Amann R. Fuchs B. M. (2008). Single-cell identification in microbial communities by improved fluorescence *in situ* hybridization techniques. Nat. Rev. Microbiol. 6, 339–348. 10.1038/nrmicro1888 18414500

[B5] Aminov R. I. (2011). Horizontal gene exchange in environmental microbiota. Front. Microbiol. 2:158. 10.3389/fmicb.2011.00158 21845185 PMC3145257

[B6] Arnold H. P. She Q. Phan H. Stedman K. Prangishvili D. Holz I.. (1999). The genetic element pSSVx of the extremely thermophilic crenarchaeon *Sulfolobus* is a hybrid between a plasmid and a virus. Mol. Microbiol. 34, 217–226. 10.1046/j.1365-2958.1999.01573.x 10564466

[B7] Atomi H. Imanaka T. Fukui T. (2012). Overview of the genetic tools in the Archaea. Front. Microbiol. 3:337. 10.3389/fmicb.2012.00337 23060865 PMC3462420

[B8] Babic M. Reskova Z. Bugala J. Cimova V. Grones P. Grones J. (2014). The Rep20 replication initiator from the pAG20 plasmid of *Acetobacter aceti*. Mol. Biotechnol. 56, 1–11. 10.1007/s12033-013-9680-6 23839792

[B9] Bao K. Cohen S. N. (2001). Terminal proteins essential for the replication of linear plasmids and chromosomes in *Streptomyces*. Genes Dev. 15, 1518–1527. 10.1101/gad.896201 11410532 PMC312717

[B10] Bao K. Cohen S. N. (2003). Recruitment of terminal protein to the ends of *Streptomyces* linear plasmids and chromosomes by a novel telomere-binding protein essential for linear DNA replication. Genes Dev. 17, 774–785. 10.1101/gad.1060303 12651895 PMC196017

[B11] Bertini A. Poirel L. Mugnier P. D. Villa L. Nordmann P. Carattoli A. (2010). Characterization and PCR-based replicon typing of resistance plasmids in *Acinetobacter baumannii*. Antimicrob. Agents Chemother. 54, 4168–4177. 10.1128/AAC.00542-10 20660691 PMC2944597

[B12] Brantl S. (2004). Plasmid replication control by antisense RNA, in Plasmid Biology, eds Phillips G. Funnell B. (Washington, DC: ASM Press), 47–62.

[B13] Brolund A. Franzen O. Melefors O. Tegmark-Wisell K. Sandegren L. (2013). Plasmidome-analysis of ESBL-producing *Escherichia coli* using conventional typing and high-throughput sequencing. PLoS ONE 8:e65793. 10.1371/journal.pone.0065793 23785449 PMC3681856

[B14] Brown C. J. Sen D. Yano H. Bauer M. L. Rogers L. M. Van der Auwera G. A.. (2013). Diverse broad-host-range plasmids from freshwater carry few accessory genes. Appl. Environ. Microbiol. 79, 7684–7695. 10.1128/AEM.02252-13 24096417 PMC3837812

[B15] Brown Kav A. Benhar I. Mizrahi I. (2013). A method for purifying high quality and high yield plasmid DNA for metagenomic and deep sequencing approaches. J. Microbiol. Methods 95, 272–279. 10.1016/j.mimet.2013.09.008 24055388

[B16] Brown Kav A. Sasson G. Jami E. Doron-Faigenboim A. Benhar I. Mizrahi I. (2012). Insights into the bovine rumen plasmidome. Proc. Natl. Acad. Sci. U.S.A. 109, 5452–5457. 10.1073/pnas.1116410109 22431592 PMC3325734

[B17] Bruand C. Ehrlich S. D. Janniere L. (1991). Unidirectional theta replication of the structurally stable *Enterococcus faecalis* plasmid pAM beta 1. EMBO J. 10, 2171–2177. 1906000 10.1002/j.1460-2075.1991.tb07752.x PMC452905

[B18] Bunikis I. Kutschan-Bunikis S. Bonde M. Bergström S. (2011). Multiplex PCR as a tool for validating plasmid content of *Borrelia burgdorferi*. J. Microbiol. Methods 86, 243–247. 10.1016/j.mimet.2011.05.004 21605603

[B19] Burmolle M. Norman A. Sorensen S. J. Hansen L. H. (2012). Sequencing of IncX-plasmids suggests ubiquity of mobile forms of a biofilm-promoting gene cassette recruited from *Klebsiella pneumoniae*. PLoS ONE 7:e41259. 10.1371/journal.pone.0041259 22844447 PMC3402527

[B20] Carattoli A. (2009). Resistance plasmid families in *Enterobacteriaceae*. Antimicrob. Agents Chemother. 53, 2227–2238. 10.1128/AAC.01707-08 19307361 PMC2687249

[B21] Carattoli A. Bertini A. Villa L. Falbo V. Hopkins K. L. Threlfall E. J. (2005). Identification of plasmids by PCR-based replicon typing. J. Microbiol. Methods 63, 219–228. 10.1016/j.mimet.2005.03.018 15935499

[B22] Casjens S. Palmer N. Van Vugt R. Huang W. M. Stevenson B. Rosa P.. (2000). A bacterial genome in flux: the twelve linear and nine circular extrachromosomal DNAs in an infectious isolate of the Lyme disease spirochete *Borrelia burgdorferi*. Mol. Microbiol. 35, 490–516. 10.1046/j.1365-2958.2000.01698.x 10672174

[B23] Casjens S. R. Mongodin E. F. Qiu W. G. Luft B. J. Schutzer S. E. Gilcrease E. B.. (2012). Genome stability of Lyme disease spirochetes: comparative genomics of *Borrelia burgdorferi* plasmids. PLoS ONE 7:e33280. 10.1371/journal.pone.0033280 22432010 PMC3303823

[B24] Cenciarini-Borde C. Courtois S. La Scola B. (2009). Nucleic acids as viability markers for bacteria detection using molecular tools. Future Microbiol. 4, 45–64. 10.2217/17460913.4.1.45 19207099

[B25] Cesareni G. Helmer-Citterich M. Castagnoli L. (1991). Control of ColE1 plasmid replication by antisense RNA. Trends Genet. 7, 230–235. 10.1016/0168-9525(91)90143-E 1887504

[B26] Cevallos M. A. Cervantes-Rivera R. Gutierrez-Rios R. M. (2008). The *repABC* plasmid family. Plasmid 60, 19–37. 10.1016/j.plasmid.2008.03.001 18433868

[B27] Chaconas G. Kobryn K. (2010). Structure, function, and evolution of linear replicons in *Borrelia*. Annu. Rev. Microbiol. 64, 185–202. 10.1146/annurev.micro.112408.134037 20536352

[B28] Chaconas G. Norris S. J. (2013). Peaceful coexistence amongst *Borrelia* plasmids: getting by with a little help from their friends? Plasmid 70, 161–167. 10.1016/j.plasmid.2013.05.002 23727020 PMC3737319

[B29] Chen C. W. Yu T. W. Lin Y. S. Kieser H. M. Hopwood D. A. (1993). The conjugative plasmid SLP2 of *Streptomyces lividans* is a 50 kb linear molecule. Mol. Microbiol. 7, 925–932. 10.1111/j.1365-2958.1993.tb01183.x 8387146

[B30] Clewell D. B. (2007). Properties of *Enterococcus faecalis* plasmid pAD1, a member of a widely disseminated family of pheromone-responding, conjugative, virulence elements encoding cytolysin. Plasmid 58, 205–227. 10.1016/j.plasmid.2007.05.001 17590438

[B31] Cobley J. G. Zerweck E. Reyes R. Mody A. Seludo-Unson J. R. Jaeger H.. (1993). Construction of shuttle plasmids which can be efficiently mobilized from *Escherichia coli* into the chromatically adapting cyanobacterium, *Fremyella diplosiphon*. Plasmid 30, 90–105. 10.1006/plas.1993.1037 8234495

[B32] Corneau N. Emond E. Lapointe G. (2004). Molecular characterization of three plasmids from *Bifidobacterium longum*. Plasmid 51, 87–100. 10.1016/j.plasmid.2003.12.003 15003705

[B33] Crowley S. Bottacini F. Mahony J. van Sinderen D. (2013). Complete genome sequence of *Lactobacillus plantarum* strain 16, a broad-spectrum antifungal-producing lactic acid bacterium. Genome Announc. 1, e00533–e00513. 10.1128/genomeA.00533-13 23908281 PMC3731835

[B34] Dealtry S. Holmsgaard P. N. Dunon V. Jechalke S. Ding G. C. Krogerrecklenfort E.. (2014). Shifts in abundance and diversity of mobile genetic elements after the introduction of diverse pesticides into an on-farm biopurification system over the course of a year. Appl. Environ. Microbiol. 80, 4012–4020. 10.1128/AEM.04016-13 24771027 PMC4054223

[B35] de Been M. Lanza V. F. de Toro M. Scharringa J. Dohmen W. Du Y.. (2014). Dissemination of cephalosporin resistance genes between *Escherichia coli* strains from farm animals and humans by specific plasmid lineages. PLoS Genet. 10:e1004776. 10.1371/journal.pgen.1004776 25522320 PMC4270446

[B36] del Solar G. Giraldo R. Ruiz-Echevarria M. J. Espinosa M. Diaz-Orejas R. (1998). Replication and control of circular bacterial plasmids. Microbiol. Mol. Biol. Rev. 62, 434–464. 9618448 10.1128/mmbr.62.2.434-464.1998 PMC98921

[B37] del Solar G. Moscoso M. Espinosa M. (1993). Rolling circle-replicating plasmids from gram-positive and gram-negative bacteria: a wall falls. Mol. Microbiol. 8, 789–796. 10.1111/j.1365-2958.1993.tb01625.x 8355606

[B38] Dijkshoorn L. Nemec A. Seifert H. (2007). An increasing threat in hospitals: multidrug-resistant *Acinetobacter baumannii*. Nat. Rev. Microbiol. 5, 939–951. 10.1038/nrmicro1789 18007677

[B39] Dunny G. M. (2007). The peptide pheromone-inducible conjugation system of *Enterococcus faecalis* plasmid pCF10: cell-cell signalling, gene transfer, complexity and evolution. Philos. Trans. R. Soc. Lond. B Biol. Sci. 362, 1185–1193. 10.1098/rstb.2007.2043 17360276 PMC2435581

[B40] Eggers C. H. Caimano M. J. Clawson M. L. Miller W. G. Samuels D. S. Radolf J. D. (2002). Identification of loci critical for replication and compatibility of a *Borrelia burgdorferi* cp32 plasmid and use of a cp32-based shuttle vector for the expression of fluorescent reporters in the lyme disease spirochaete. Mol. Microbiol. 43, 281–295. 10.1046/j.1365-2958.2002.02758.x 11985709

[B41] Eikmeyer F. Hadiati A. Szczepanowski R. Wibberg D. Schneiker-Bekel S. Rogers L. M.. (2012). The complete genome sequences of four new IncN plasmids from wastewater treatment plant effluent provide new insights into IncN plasmid diversity and evolution. Plasmid 68, 13–24. 10.1016/j.plasmid.2012.01.011 22326849

[B42] Elsaied H. Stokes H. W. Kitamura K. Kurusu Y. Kamagata Y. Maruyama A. (2011). Marine integrons containing novel integrase genes, attachment sites, attI, and associated gene cassettes in polluted sediments from Suez and Tokyo Bays. ISME J. 5, 1162–1177. 10.1038/ismej.2010.208 21248857 PMC3146285

[B43] Encinas D. Garcillan-Barcia M. P. Santos-Merino M. Delaye L. Moya A. de la Cruz F. (2014). Plasmid conjugation from proteobacteria as evidence for the origin of xenologous genes in cyanobacteria. J. Bacteriol. 196, 1551–1559. 10.1128/JB.01464-13 24509315 PMC3993370

[B44] Espinosa M. Cohen S. Couturier M. Del Solar G. Diaz-Orejas R. Giraldo R. (2000). Plasmid replication and copy number control, in The Horizontal Gene Pool: Bacterial Plasmids and Gene Spread, ed Thomas C. M. (Amsterdam: Harwood Academic Publishers), 1–47.

[B45] Evans B. A. Amyes S. G. (2014). OXA beta-lactamases. Clin. Microbiol. Rev. 27, 241–263. 10.1128/CMR.00117-13 24696435 PMC3993105

[B46] Fraser C. M. Casjens S. Huang W. M. Sutton G. G. Clayton R. Lathigra R.. (1997). Genomic sequence of a Lyme disease spirochaete, *Borrelia burgdorferi*. Nature 390, 580–586. 10.1038/37551 9403685

[B47] Frost L. Leplae R. Summers A. Toussaint A. (2005). Mobile genetic elements: the agents of open source evolution. Nat. Rev. Microbiol. 3, 722–732. 10.1038/nrmicro1235 16138100

[B48] Frost L. S. Koraimann G. (2010). Regulation of bacterial conjugation: balancing opportunity with adversity. Future Microbiol. 5, 1057–1071. 10.2217/fmb.10.70 20632805

[B49] Fukao M. Oshima K. Morita H. Toh H. Suda W. Kim S. W.. (2013). Genomic analysis by deep sequencing of the probiotic *Lactobacillus brevis* KB290 harboring nine plasmids reveals genomic stability. PLoS ONE 8:e60521. 10.1371/journal.pone.0060521 23544154 PMC3609814

[B50] Fukaya M. Okumura H. Masai H. Uozumi T. Beppu T. (1985). Construction of new shuttle vectors for *Acetobacter*. Agric. Biol. Chem. 49, 2083–2090 10.1271/bbb1961.49.2083

[B51] Funnell B. Phillips G. (2004). Preface, in Plasmid Biology, eds Funnell B. Phillips G. (Washington, DC: ASM Press), xi.

[B52] Garcillán-Barcia M. P. Alvarado A. de la Cruz F. (2011). Identification of bacterial plasmids based on mobility and plasmid population biology. FEMS Microbiol. Rev. 35, 936–956. 10.1111/j.1574-6976.2011.00291.x 21711366

[B53] Garcillán-Barcia M. P. Francia M. V. de la Cruz F. (2009). The diversity of conjugative relaxases and its application in plasmid classification. FEMS Microbiol. Rev. 33, 657–687. 10.1111/j.1574-6976.2009.00168.x 19396961

[B54] Giraldo R. Fernandez-Tresguerres M. E. (2004). Twenty years of the pPS10 replicon: insights on the molecular mechanism for the activation of DNA replication in iteron-containing bacterial plasmids. Plasmid 52, 69–83. 10.1016/j.plasmid.2004.06.002 15336485

[B55] Goessweiner-Mohr N. Arends K. Keller W. Grohmann E. (2013). Conjugative type IV secretion systems in Gram-positive bacteria. Plasmid 70, 289–302. 10.1016/j.plasmid.2013.09.005 24129002 PMC3913187

[B56] Golden S. S. Sherman L. A. (1983). A hybrid plasmid is a stable cloning vector for the cyanobacterium *Anacystis nidulans* R2. J. Bacteriol. 155, 966–972. 6309751 10.1128/jb.155.3.966-972.1983 PMC217787

[B58] Greve B. Jensen S. Brügger K. Zillig W. Garrett R. A. (2004). Genomic comparison of archaeal conjugative plasmids from *Sulfolobus*. Archaea 1, 231–239. 10.1155/2004/151926 15810432 PMC2685578

[B59] Grones P. Grones J. (2012). Characterization of the theta replication plasmid pGR7 from *Acetobacter aceti* CCM 3610. Res. Microbiol. 163, 419–426. 10.1016/j.resmic.2012.07.002 22842078

[B60] Guglielmetti S. Mora D. Parini C. (2007). Small rolling circle plasmids in *Bacillus subtilis* and related species: organization, distribution, and their possible role in host physiology. Plasmid 57, 245–264. 10.1016/j.plasmid.2006.09.002 17064773

[B61] Guglielmini J. Quintais L. Garcillán-Barcia M. P. de la Cruz F. Rocha E. P. (2011). The repertoire of ICE in prokaryotes underscores the unity, diversity, and ubiquity of conjugation. PLoS Genet. 7:e1002222. 10.1371/journal.pgen.1002222 21876676 PMC3158045

[B62] Heuer H. Binh C. T. Jechalke S. Kopmann C. Zimmerling U. Krogerrecklenfort E.. (2012). IncP-1epsilon plasmids are important vectors of antibiotic resistance genes in agricultural systems: diversification driven by class 1 integron gene cassettes. Front. Microbiol. 3:2. 10.3389/fmicb.2012.00002 22279444 PMC3260659

[B63] Heuer H. Smalla K. (2007). Horizontal gene transfer between bacteria. Environ. Biosafety Res. 6, 3–13. 10.1051/ebr:2007034 17961477

[B64] Heuer H. Smalla K. (2012). Plasmids foster diversification and adaptation of bacterial populations in soil. FEMS Microbiol. Rev. 36, 1083–1104. 10.1111/j.1574-6976.2012.00337.x 22393901

[B65] Hou C. Wang Q. Zeng X. Yang F. Zhang J. Liu H.. (2014). Complete genome sequence of *Lactobacillus reuteri* I5007, a probiotic strain isolated from healthy piglet. J. Biotechnol. 179, 63–64. 10.1016/j.jbiotec.2014.03.019 24685642

[B66] Huang C. H. Tsai H. H. Tsay Y. G. Chien Y. N. Wang S. L. Cheng M. Y.. (2007). The telomere system of the *Streptomyces* linear plasmid SCP1 represents a novel class. Mol. Microbiol. 63, 1710–1718. 10.1111/j.1365-2958.2007.05616.x 17367390

[B67] Jayashree S. Pooja S. Pushpanathan M. Vishnu U. Sankarasubramanian J. Rajendhran J.. (2013). Genome sequence of *Lactobacillus fermentum* strain MTCC 8711, a probiotic bacterium isolated from yogurt. Genome Announc. 1, e00770–e00713. 10.1128/genomeA.00770-13 24072868 PMC3784788

[B68] Jensen L. B. Garcia-Migura L. Valenzuela A. J. Løhr M. Hasman H. Aarestrup F. M. (2010a). A classification system for plasmids from enterococci and other Gram-positive bacteria. J. Microbiol. Methods 80, 25–43. 10.1016/j.mimet.2009.10.012 19879906

[B69] Jensen S. O. Apisiridej S. Kwong S. M. Yang Y. H. Skurray R. A. Firth N. (2010b). Analysis of the prototypical *Staphylococcus aureus* multiresistance plasmid pSK1. Plasmid 64, 135–142. 10.1016/j.plasmid.2010.06.001 20547176

[B70] Jeon C. O. Park M. Ro H. S. Park W. Madsen E. L. (2006). The naphthalene catabolic (*nag*) genes of *Polaromonas naphthalenivorans* CJ2: evolutionary implications for two gene clusters and novel regulatory control. Appl. Environ. Microbiol. 72, 1086–1095. 10.1128/AEM.72.2.1086-1095.2006 16461653 PMC1392936

[B71] Jeon C. O. Park W. Padmanabhan P. Derito C. Snape J. R. Madsen E. L. (2003). Discovery of a bacterium, with distinctive dioxygenase, that is responsible for *in situ* biodegradation in contaminated sediment. Proc. Natl. Acad. Sci. U.S.A. 100, 13591–13596. 10.1073/pnas.1735529100 14597712 PMC263858

[B72] Johnson T. J. Nolan L. K. (2009). Pathogenomics of the virulence plasmids of *Escherichia coli*. Microbiol. Mol. Biol. Rev. 73, 750–774. 10.1128/MMBR.00015-09 19946140 PMC2786578

[B73] Jones B. V. Marchesi J. R. (2007). Transposon-aided capture (TRACA) of plasmids resident in the human gut mobile metagenome. Nat. Methods 4, 55–61. 10.1038/nmeth964 17128268

[B74] Jorgensen T. S. Xu Z. Hansen M. A. Sorensen S. J. Hansen L. H. (2014). Hundreds of circular novel plasmids and DNA elements identified in a rat cecum metamobilome. PLoS ONE 9:e87924. 10.1371/journal.pone.0087924 24503942 PMC3913684

[B75] Joshua C. J. Perez L. D. Keasling J. D. (2013). Functional characterization of the origin of replication of pRN1 from *Sulfolobus islandicus* REN1H1. PLoS ONE 8:e84664. 10.1371/journal.pone.0084664 24376833 PMC3869888

[B76] Kendall K. J. Cohen S. N. (1988). Complete nucleotide sequence of the *Streptomyces lividans* plasmid pIJ101 and correlation of the sequence with genetic properties. J. Bacteriol. 170, 4634–4651. 3170481 10.1128/jb.170.10.4634-4651.1988 PMC211503

[B77] Khan S. A. (2005). Plasmid rolling-circle replication: highlights of two decades of research. Plasmid 53, 126–136. 10.1016/j.plasmid.2004.12.008 15737400

[B78] Kristiansson E. Fick J. Janzon A. Grabic R. Rutgersson C. Weijdegård B.. (2011). Pyrosequencing of antibiotic-contaminated river sediments reveals high levels of resistance and gene transfer elements. PLoS ONE 6:e17038. 10.1371/journal.pone.0017038 21359229 PMC3040208

[B79] Krüger R. Rakowski S. A. Filutowicz M. (2004). Participating elements in the replication of iteron-containing plasmids, in Plasmid Biology, eds Phillips G. Funnell B. (Washington, DC: ASM Press), 25–45.

[B80] Labidi A. David H. L. Roulland-Dussoix D. (1985). Restriction endonuclease mapping and cloning of *Mycobacterium fortuitum* var. *fortuitum* plasmid pAL5000. Ann. Inst. Pasteur Microbiol. 136B, 209–215. 10.1016/S0769-2609(85)80045-4 3002238

[B81] Lambert G. Carr N. (1983). A restriction map of plasmid pDC1 from the filamentous cyanobacterium *Nostoc* sp. MAC PCC 8009. Plasmid 10, 196–198. 10.1016/0147-619X(83)90072-0 6314412

[B82] Lawley T. Wilkins B. M. Frost L. S. (2004). Bacterial conjugation in Gram-negative bacteria, in Plasmid Biology, eds Phillips G. Funnell B. (Washington, DC: ASM Press), 203–226.

[B83] Lawrence J. G. Ochman H. (1998). Molecular archaeology of the *Escherichia coli* genome. Proc. Natl. Acad. Sci. U.S.A. 95, 9413–9417. 10.1073/pnas.95.16.9413 9689094 PMC21352

[B84] Le Dantec C. Winter N. Gicquel B. Vincent V. Picardeau M. (2001). Genomic sequence and transcriptional analysis of a 23-kilobase mycobacterial linear plasmid: evidence for horizontal transfer and identification of plasmid maintenance systems. J. Bacteriol. 183, 2157–2164. 10.1128/JB.183.7.2157-2164.2001 11244052 PMC95119

[B85] Lee J. H. O'Sullivan D. J. (2006). Sequence analysis of two cryptic plasmids from *Bifidobacterium longum* DJO10A and construction of a shuttle cloning vector. Appl. Environ. Microbiol. 72, 527–535. 10.1128/AEM.72.1.527-535.2006 16391088 PMC1352255

[B87] Leigh J. A. Albers S. V. Atomi H. Allers T. (2011). Model organisms for genetics in the domain *Archaea*: methanogens, halophiles, *Thermococcales* and *Sulfolobales*. FEMS Microbiol. Rev. 35, 577–608. 10.1111/j.1574-6976.2011.00265.x 21265868

[B88] Liu M. A. Kwong S. M. Jensen S. O. Brzoska A. J. Firth N. (2013). Biology of the staphylococcal conjugative multiresistance plasmid pSK41. Plasmid 70, 42–51. 10.1016/j.plasmid.2013.02.001 23415796

[B89] Lozano C. García-Migura L. Aspiroz C. Zarazaga M. Torres C. Aarestrup F. M. (2012). Expansion of a plasmid classification system for Gram-positive bacteria and determination of the diversity of plasmids in *Staphylococcus aureus* strains of human, animal, and food origins. Appl. Environ. Microbiol. 78, 5948–5955. 10.1128/AEM.00870-12 22685157 PMC3406130

[B90] Luo Y. R. Kang S. G. Kim S. J. Kim M. R. Li N. Lee J. H.. (2012). Genome sequence of benzo(a)pyrene-degrading bacterium *Novosphingobium pentaromativorans* US6-1. J. Bacteriol. 194, 907. 10.1128/JB.06476-11 22275104 PMC3272951

[B91] Masai E. Sugiyama K. Iwashita N. Shimizu S. Hauschild J. E. Hatta T.. (1997). The *bphDEF* meta-cleavage pathway genes involved in biphenyl/polychlorinated biphenyl degradation are located on a linear plasmid and separated from the initial *bphACB* genes in *Rhodococcus* sp. strain RHA1. Gene 187, 141–149. 10.1016/S0378-1119(96)00748-2 9073078

[B92] McLeod M. P. Warren R. L. Hsiao W. W. Araki N. Myhre M. Fernandes C.. (2006). The complete genome of *Rhodococcus* sp. RHA1 provides insights into a catabolic powerhouse. Proc. Natl. Acad. Sci. U.S.A. 103, 15582–15587. 10.1073/pnas.0607048103 17030794 PMC1622865

[B93] Metcalf W. W. Zhang J. K. Apolinario E. Sowers K. R. Wolfe R. S. (1997). A genetic system for *Archaea* of the genus *Methanosarcina*: liposome-mediated transformation and construction of shuttle vectors. Proc. Natl. Acad. Sci. U.S.A. 94, 2626–2631. 10.1073/pnas.94.6.2626 9122246 PMC20139

[B94] Miller T. R. Delcher A. L. Salzberg S. L. Saunders E. Detter J. C. Halden R. U. (2010). Genome sequence of the dioxin-mineralizing bacterium *Sphingomonas wittichii* RW1. J. Bacteriol. 192, 6101–6102. 10.1128/JB.01030-10 20833805 PMC2976435

[B95] Miyazaki R. Sato Y. Ito M. Ohtsubo Y. Nagata Y. Tsuda M. (2006). Complete nucleotide sequence of an exogenously isolated plasmid, pLB1, involved in gamma-hexachlorocyclohexane degradation. Appl. Environ. Microbiol. 72, 6923–6933. 10.1128/AEM.01531-06 16963556 PMC1636184

[B96] Musovic S. Dechesne A. Sørensen J. Smets B. F. (2010). Novel assay to assess permissiveness of a soil microbial community toward receipt of mobile genetic elements. Appl. Environ. Microbiol. 76, 4813–4818. 10.1128/AEM.02713-09 20511430 PMC2901734

[B97] Nagata Y. Kamakura M. Endo R. Miyazaki R. Ohtsubo Y. Tsuda M. (2006). Distribution of gamma-hexachlorocyclohexane-degrading genes on three replicons in *Sphingobium japonicum* UT26. FEMS Microbiol. Lett. 256, 112–118. 10.1111/j.1574-6968.2005.00096.x 16487327

[B98] Nagata Y. Natsui S. Endo R. Ohtsubo Y. Ichikawa N. Ankai A.. (2011). Genomic organization and genomic structural rearrangements of *Sphingobium japonicum* UT26, an archetypal γ-hexachlorocyclohexane-degrading bacterium. Enzyme Microb. Technol. 49, 499–508. 10.1016/j.enzmictec.2011.10.005 22142724

[B99] Nagata Y. Ohtsubo Y. Endo R. Ichikawa N. Ankai A. Oguchi A.. (2010). Complete genome sequence of the representative γ-hexachlorocyclohexane-degrading bacterium *Sphingobium japonicum* UT26. J. Bacteriol. 192, 5852–5853. 10.1128/JB.00961-10 20817768 PMC2953701

[B100] Nishida H. (2012). Comparative analyses of base compositions, DNA sizes, and dinucleotide frequency profiles in archaeal and bacterial chromosomes and plasmids. Int. J. Evol. Biol. 2012:342482. 10.1155/2012/342482 22536540 PMC3321278

[B101] Norberg P. Bergstrom M. Hermansson M. (2014). Complete nucleotide sequence and analysis of two conjugative broad host range plasmids from a marine microbial biofilm. PLoS ONE 9:e92321. 10.1371/journal.pone.0092321 24647540 PMC3960245

[B102] Okumura H. Uozumi T. Beppu T. (1985). Construction of plasmid vectors and a genetic transformation system for *Acetobacter aceti*. Agric. Biol. Chem. 49, 1011–1017 10.1271/bbb1961.49.1011

[B103] Oliveira C. S. Moura A. Henriques I. Brown C. J. Rogers L. M. Top E. M.. (2013). Comparative genomics of IncP-1epsilon plasmids from water environments reveals diverse and unique accessory genetic elements. Plasmid 70, 412–419. 10.1016/j.plasmid.2013.06.002 23831558

[B104] Palmer K. L. Kos V. N. Gilmore M. S. (2010). Horizontal gene transfer and the genomics of enterococcal antibiotic resistance. Curr. Opin. Microbiol. 13, 632–639. 10.1016/j.mib.2010.08.004 20837397 PMC2955785

[B105] Pandza S. Biukovic G. Paravic A. Dadbin A. Cullum J. Hranueli D. (1998). Recombination between the linear plasmid pPZG101 and the linear chromosome of *Streptomyces rimosus* can lead to exchange of ends. Mol. Microbiol. 28, 1165–1176. 10.1046/j.1365-2958.1998.00877.x 9680206

[B106] Park M. S. Shin D. W. Lee K. H. Ji G. E. (1999). Sequence analysis of plasmid pKJ50 from *Bifidobacterium longum*. Microbiology 145(Pt 3), 585–592. 10.1099/13500872-145-3-585 10217492

[B107] Partridge S. R. (2011). Analysis of antibiotic resistance regions in Gram-negative bacteria. FEMS Microbiol. Rev. 35, 820–855. 10.1111/j.1574-6976.2011.00277.x 21564142

[B108] Paterson D. L. (2006). Resistance in gram-negative bacteria: *Enterobacteriaceae*. Am. J. Infect. Control 34, S20–S28. discussion: S64–S73. 10.1016/j.ajic.2006.05.238 16813978

[B109] Petersen J. M. Zielinski F. U. Pape T. Seifert R. Moraru C. Amann R.. (2011). Hydrogen is an energy source for hydrothermal vent symbioses. Nature 476, 176–180. 10.1038/nature10325 21833083

[B110] Pinto U. M. Pappas K. M. Winans S. C. (2012). The ABCs of plasmid replication and segregation. Nat. Rev. Microbiol. 10, 755–765. 10.1038/nrmicro2882 23070556

[B111] Poehlein A. Kusian B. Friedrich B. Daniel R. Bowien B. (2011). Complete genome sequence of the type strain *Cupriavidus necator* N-1. J. Bacteriol. 193, 5017. 10.1128/JB.05660-11 21742890 PMC3165677

[B112] Polisky B. (1988). ColE1 replication control circuitry: sense from antisense. Cell 55, 929–932. 10.1016/0092-8674(88)90235-8 2462471

[B114] Rakowski S. A. Filutowicz M. (2013). Plasmid R6K replication control. Plasmid 69, 231–242. 10.1016/j.plasmid.2013.02.003 23474464 PMC3691012

[B115] Ravin N. V. (2011). N15: the linear phage-plasmid. Plasmid 65, 102–109. 10.1016/j.plasmid.2010.12.004 21185326

[B116] Rocha E. P. Danchin A. (2002). Base composition bias might result from competition for metabolic resources. Trends Genet. 18, 291–294. 10.1016/S0168-9525(02)02690-2 12044357

[B117] Romine M. F. Stillwell L. C. Wong K. K. Thurston S. J. Sisk E. C. Sensen C.. (1999). Complete sequence of a 184-kilobase catabolic plasmid from *Sphingomonas aromaticivorans* F199. J. Bacteriol. 181, 1585–1602. 10049392 10.1128/jb.181.5.1585-1602.1999 PMC93550

[B118] Rosvoll T. C. Pedersen T. Sletvold H. Johnsen P. J. Sollid J. E. Simonsen G. S.. (2010). PCR-based plasmid typing in *Enterococcus faecium* strains reveals widely distributed pRE25-, pRUM-, pIP501- and pHTbeta-related replicons associated with glycopeptide resistance and stabilizing toxin-antitoxin systems. FEMS Immunol. Med. Microbiol. 58, 254–268. 10.1111/j.1574-695X.2009.00633.x 20015231

[B119] Sekine M. Tanikawa S. Omata S. Saito M. Fujisawa T. Tsukatani N.. (2006). Sequence analysis of three plasmids harboured in *Rhodococcus erythropolis* strain PR4. Environ. Microbiol. 8, 334–346. 10.1111/j.1462-2920.2005.00899.x 16423019

[B120] Sen D. Van der Auwera G. A. Rogers L. M. Thomas C. M. Brown C. J. Top E. M. (2011). Broad-host-range plasmids from agricultural soils have IncP-1 backbones with diverse accessory genes. Appl. Environ. Microbiol. 77, 7975–7983. 10.1128/AEM.05439-11 21948829 PMC3209000

[B121] Sentchilo V. Mayer A. P. Guy L. Miyazaki R. Green Tringe S. Barry K.. (2013). Community-wide plasmid gene mobilization and selection. ISME J. 7, 1173–1186. 10.1038/ismej.2013.13 23407308 PMC3660673

[B122] Seto M. Kimbara K. Shimura M. Hatta T. Fukuda M. Yano K. (1995). A novel transformation of polychlorinated biphenyls by *Rhodococcus* sp. strain RHA1. Appl. Environ. Microbiol. 61, 3353–3358. 16535122 10.1128/aem.61.9.3353-3358.1995 PMC1388576

[B123] Shareck J. Choi Y. Lee B. Miguez C. B. (2004). Cloning vectors based on cryptic plasmids isolated from lactic acid bacteria: their characteristics and potential applications in biotechnology. Crit. Rev. Biotechnol. 24, 155–208. 10.1080/07388550490904288 15707158

[B124] She Q. Phan H. Garrett R. A. Albers S. V. Stedman K. M. Zillig W. (1998). Genetic profile of pNOB8 from *Sulfolobus*: the first conjugative plasmid from an archaeon. Extremophiles 2, 417–425. 10.1007/s007920050087 9827331

[B125] Shimizu S. Kobayashi H. Masai E. Fukuda M. (2001). Characterization of the 450-kb linear plasmid in a polychlorinated biphenyl degrader, *Rhodococcus* sp. strain RHA1. Appl. Environ. Microbiol. 67, 2021–2028. 10.1128/AEM.67.5.2021-2028.2001 11319076 PMC92831

[B126] Shintani M. Matsui K. Inoue J. Hosoyama A. Ohji S. Yamazoe A.. (2014a). Single-cell analyses revealed transfer ranges of IncP-1, IncP-7, and IncP-9 plasmids in a soil bacterial community. Appl. Environ. Microbiol. 80, 138–145. 10.1128/AEM.02571-13 24141122 PMC3911017

[B127] Shintani M. Nojiri H. (2013). Mobile genetic elements (MGEs) carrying catabolic genes, in Management of Microbial Resources in the Environment, eds Malik A. Grohmann E. Alves M. (Dordrecht: Springer), 167–214 10.1007/978-94-007-5931-2_8

[B128] Shintani M. Ohtsubo Y. Fukuda K. Hosoyama A. Ohji S. Yamazoe A.. (2014b). Complete genome sequence of the thermophilic polychlorinated biphenyl degrader *Geobacillus* sp. strain JF8 (NBRC 109937). Genome Announc 2, e01213–13. 10.1128/genomeA.01213-13 24459274 PMC3900906

[B129] Shintani M. Takahashi Y. Yamane H. Nojiri H. (2010). The behavior and significance of degradative plasmids belonging to Inc groups in *Pseudomonas* within natural environments and microcosms. Microbes Environ. 25, 253–265. 10.1264/jsme2.ME10155 21576880

[B130] Smalla K. Sobecky P. A. (2002). The prevalence and diversity of mobile genetic elements in bacterial communities of different environmental habitats: insights gained from different methodological approaches. FEMS Microbiol. Ecol. 42, 165–175. 10.1111/j.1574-6941.2002.tb01006.x 19709276

[B131] Smillie C. Garcillán-Barcia M. P. Francia M. V. Rocha E. P. de la Cruz F. (2010). Mobility of plasmids. Microbiol. Mol. Biol. Rev. 74, 434–452. 10.1128/MMBR.00020-10 20805406 PMC2937521

[B132] Smith M. C. Thomas C. D. (2004). An accessory protein is required for relaxosome formation by small staphylococcal plasmids. J. Bacteriol. 186, 3363–3373. 10.1128/JB.186.11.3363-3373.2004 15150221 PMC415746

[B133] Sobecky P. A. Hazen T. H. (2009). Horizontal gene transfer and mobile genetic elements in marine systems. Methods Mol. Biol. 532, 435–453. 10.1007/978-1-60327-853-9_25 19271200

[B134] Soler N. Justome A. Quevillon-Cheruel S. Lorieux F. Le Cam E. Marguet E.. (2007). The rolling-circle plasmid pTN1 from the hyperthermophilic archaeon *Thermococcus nautilus*. Mol. Microbiol. 66, 357–370. 10.1111/j.1365-2958.2007.05912.x 17784911

[B135] Song X. Sun J. Mikalsen T. Roberts A. P. Sundsfjord A. (2013). Characterisation of the plasmidome within *Enterococcus faecalis* isolated from marginal periodontitis patients in Norway. PLoS ONE 8:e62248. 10.1371/journal.pone.0062248 23646122 PMC3639998

[B136] Sorvig E. Mathiesen G. Naterstad K. Eijsink V. G. Axelsson L. (2005). High-level, inducible gene expression in *Lactobacillus sakei* and *Lactobacillus plantarum* using versatile expression vectors. Microbiology 151, 2439–2449. 10.1099/mic.0.28084-0 16000734

[B137] Sota M. Top E. (2008). Horizontal gene transfer mediated by plasmids, in Plasmids: Current Research and Future Trends, ed Lipps G. (Norfolk, VA: Caister Academic Press; Horizon Scientific Press), 111–181.

[B139] Stillwell L. C. Thurston S. J. Schneider R. P. Romine M. F. Fredrickson J. K. Saffer J. D. (1995). Physical mapping and characterization of a catabolic plasmid from the deep-subsurface bacterium *Sphingomonas* sp. strain F199. J. Bacteriol. 177, 4537–4539. 7635838 10.1128/jb.177.15.4537-4539.1995 PMC177210

[B140] Straley S. C. Plano G. V. Skrzypek E. Haddix P. L. Fields K. A. (1993). Regulation by Ca^2+^ in the *Yersinia* low-Ca^2+^ response. Mol. Microbiol. 8, 1005–1010. 10.1111/j.1365-2958.1993.tb01644.x 8361348

[B141] Taton A. Unglaub F. Wright N. E. Zeng W. Y. Paz-Yepes J. Brahamsha B.. (2014). Broad-host-range vector system for synthetic biology and biotechnology in cyanobacteria. Nucleic Acids Res. 42:e136. 10.1093/nar/gku673 25074377 PMC4176158

[B156] Tauch A. Bischoff N. Brune I. Kalinowski J. (2003a). Insights into the genetic organization of the *Corynebacterium diphtheriae* erythromycin resistance plasmid pNG2 deduced from its complete nucleotide sequence. Plasmid 49, 63–74. 10.1016/S0147-619X(02)00115-4 12584002

[B157] Tauch A. Pühler A. Kalinowski J. Thierbach G. (2003b). Plasmids in *Corynebacterium glutamicum* and their molecular classification by comparative genomics. J. Biotechnol. 104, 27–40. 10.1016/S0168-1656(03)00157-3 12948627

[B142] Taylor D. E. Gibreel A. Lawley T. D. Tracz D. M. (2004). Anitibiotic resistance plasmids, in Plasmid Biology, eds Phillips G. Funnell B. (Washington, DC: ASM Press), 473–491.

[B143] Thomas C. M. Haines A. S. (2004). Plasmids of the genus *Pseudomonas*, in Pseudomonas, ed Ramos J. L. (New York, NY: Plenum Publishing Corporation), 197–231.

[B144] Tilly K. Checroun C. Rosa P. A. (2012). Requirements for *Borrelia burgdorferi* plasmid maintenance. Plasmid 68, 1–12. 10.1016/j.plasmid.2012.01.009 22289894 PMC3367046

[B145] Torres M. J. Rubia M. I. Bedmar E. J. Delgado M. J. (2011). Denitrification in *Sinorhizobium meliloti*. Biochem. Soc. Trans. 39, 1886–1889. 10.1042/BST20110733 22103545

[B146] Tumbula D. L. Bowen T. L. Whitman W. B. (1997). Characterization of pURB500 from the archaeon *Methanococcus maripaludis* and construction of a shuttle vector. J. Bacteriol. 179, 2976–2986. 9139917 10.1128/jb.179.9.2976-2986.1997 PMC179063

[B147] Udo E. E. Grubb W. B. (1991). A new incompatibility group plasmid in *Staphylococcus aureus*. FEMS Microbiol. Lett. 62, 33–36. 10.1111/j.1574-6968.1991.tb04412.x 2032622

[B148] Ventura M. Canchaya C. Tauch A. Chandra G. Fitzgerald G. F. Chater K. F.. (2007). Genomics of *Actinobacteria*: tracing the evolutionary history of an ancient phylum. Microbiol. Mol. Biol. Rev. 71, 495–548. 10.1128/MMBR.00005-07 17804669 PMC2168647

[B149] Wagner M. Haider S. (2012). New trends in fluorescence *in situ* hybridization for identification and functional analyses of microbes. Curr. Opin. Biotechnol. 23, 96–102. 10.1016/j.copbio.2011.10.010 22079351

[B150] Walton D. K. Gendel S. M. Atherly A. G. (1992). Nucleotide sequence of the replication region of the *Nostoc* PCC 7524 plasmid pDU1. Nucleic Acids Res. 20:4660. 10.1093/nar/20.17.4660 1408769 PMC334200

[B151] Wang J. Stephan R. Karczmarczyk M. Yan Q. Hachler H. Fanning S. (2013). Molecular characterization of bla ESBL-harboring conjugative plasmids identified in multi-drug resistant *Escherichia coli* isolated from food-producing animals and healthy humans. Front. Microbiol. 4:188. 10.3389/fmicb.2013.00188 23874325 PMC3708134

[B152] Xiong J. Alexander D. C. Ma J. H. Deraspe M. Low D. E. Jamieson F. B.. (2013). Complete sequence of pOZ176, a 500-kilobase IncP-2 plasmid encoding IMP-9-mediated carbapenem resistance, from outbreak isolate *Pseudomonas aeruginosa* 96. Antimicrob. Agents Chemother. 57, 3775–3782. 10.1128/AAC.00423-13 23716048 PMC3719692

[B153] Yagi J. M. Sims D. Brettin T. Bruce D. Madsen E. L. (2009). The genome of *Polaromonas naphthalenivorans* strain CJ2, isolated from coal tar-contaminated sediment, reveals physiological and metabolic versatility and evolution through extensive horizontal gene transfer. Environ. Microbiol. 11, 2253–2270. 10.1111/j.1462-2920.2009.01947.x 19453698

[B154] Yamashita A. Sekizuka T. Kuroda M. (2014). Characterization of antimicrobial resistance dissemination across plasmid communities classified by network analysis. Pathogens 3, 356–376. 10.3390/pathogens3020356 25437804 PMC4243450

[B155] Zhang T. Zhang X. X. Ye L. (2011). Plasmid metagenome reveals high levels of antibiotic resistance genes and mobile genetic elements in activated sludge. PLoS ONE 6:e26041. 10.1371/journal.pone.0026041 22016806 PMC3189950

